# Z-disc protein CHAPb induces cardiomyopathy and contractile dysfunction in the postnatal heart

**DOI:** 10.1371/journal.pone.0189139

**Published:** 2017-12-05

**Authors:** Willemijn van Eldik, Brigit den Adel, Jantine Monshouwer-Kloots, Daniela Salvatori, Saskia Maas, Ingeborg van der Made, Esther E. Creemers, Derk Frank, Norbert Frey, Nicky Boontje, Jolanda van der Velden, Paul Steendijk, Christine Mummery, Robert Passier, Abdelaziz Beqqali

**Affiliations:** 1 Department of Anatomy and Embryology, Leiden University Medical Center, Leiden, The Netherlands; 2 Interuniversity Cardiology Institute of the Netherlands (ICIN), Utrecht, The Netherlands; 3 Central Laboratory Animal Facility, Leiden University Medical Center, Leiden, The Netherlands; 4 Department of Experimental Cardiology, Academic Medical Center, Amsterdam, The Netherlands; 5 Department of Cardiology and Angiology, Universitätsklinikum Schleswig-Holstein (UKSH), University of Kiel, Kiel, Germany; 6 Laboratory for Physiology, Institute for Cardiovascular Research, VU University Medical Center, Amsterdam, The Netherlands; 7 Department of Cardiology, Leiden University Medical Center, Leiden, The Netherlands; Scuola Superiore Sant'Anna, ITALY

## Abstract

**Aims:**

The Z-disc is a crucial structure of the sarcomere and is implicated in mechanosensation/transduction. Dysregulation of Z-disc proteins often result in cardiomyopathy. We have previously shown that the Z-disc protein Cytoskeletal Heart-enriched Actin-associated Protein (CHAP) is essential for cardiac and skeletal muscle development. Furthermore, the CHAP gene has been associated with atrial fibrillation in humans. Here, we studied the misregulated expression of CHAP isoforms in heart disease.

**Methods and results:**

Mice that underwent transverse aortic constriction and calcineurin transgenic (Tg) mice, both models of experimental heart failure, displayed a significant increase in cardiac expression of fetal isoform CHAPb. To investigate whether increased expression of CHAPb postnatally is sufficient to induce cardiomyopathy, we generated CHAPb Tg mice under the control of the cardiac-specific αMHC promoter. CHAPb Tg mice displayed cardiac hypertrophy, interstitial fibrosis and enlargement of the left atrium at three months, which was more pronounced at the age of six months. Hypertrophy and fibrosis were confirmed by evidence of activation of the hypertrophic gene program (*Nppa*, *Nppb*, *Myh7*) and increased collagen expression, respectively. Connexin40 and 43 were downregulated in the left atrium, which was associated with delayed atrioventricular conduction. Tg hearts displayed both systolic and diastolic dysfunction partly caused by impaired sarcomere function evident from a reduced force generating capacity of single cardiomyocytes. This co-incided with activation of the actin signalling pathway leading to the formation of stress fibers.

**Conclusion:**

This study demonstrated that the fetal isoform CHAPb initiates progression towards cardiac hypertrophy, which is accompanied by delayed atrioventricular conduction and diastolic dysfunction. Moreover, CHAP may be a novel therapeutic target or candidate gene for screening in cardiomyopathies and atrial fibrillation.

## Introduction

Heart disease is a life-threatening and complex multifactorial disease, with both genetic and environmental risk factors contributing to the onset and progression of the disease. Cardiac hypertrophy, indicated by an increase in heart size and weight, is an important determinant of heart disease. Although pathological cardiac hypertrophy is initially a compensatory mechanism in response to stressful conditions such as cardiomyocyte loss or increased pressure overload, eventually it leads to impaired cardiac function and progression to heart failure [1–3]. In addition to the adaptive hypertrophic response of the heart, cardiac hypertrophy can also be caused by intrinsic genetic defects, affecting sarcomeric and cytoskeletal proteins directly, such as in hypertrophic and dilated cardiomyopathy (HCM and DCM). Despite the increase in genetic associations with familial cardiomyopathies, our understanding of molecular regulation and signal pathways involved in heart disease is still limited.

Nearly all genes produce multiple isoforms of mRNAs by alternative splicing or the use of alternative transcription start sites. These are important mechanisms for generating a variety of proteins with distinct functions from a single gene. Recently it has been shown that transcriptome-wide changes in mRNA isoforms of sarcomeric genes occur in several forms of human heart disease, including aortic stenosis [4, 5]. However, it is unknown to what extent the misregulated isoform expression of a single gene can contribute to heart disease.

Previously, we described a novel protein, named Cytoskeletal Heart-enriched Actin-associated Protein (CHAP) [6, 7], which is expressed in striated muscle at the Z-disc of sarcomeres. We identified two isoforms of CHAP (encoded by a single gene), which were differentially expressed during embryonic and postnatal development. CHAPb is expressed during early cardiac and skeletal muscle development and is downregulated in adult tissues, whereas CHAPa is predominantly expressed in adult heart and skeletal muscle. Furthermore, CHAPa, the longest isoform (978 amino acids (aa)) contains an N-terminal PDZ domain, as well as a nuclear localization signal (NLS) while CHAPb isoform (749 aa) lacks the PDZ domain. Zebrafish and chick orthologues of CHAP, which only express the CHAPa isoform, are present in the heart during embryonic development from the cardiac crescent stage onwards, and in later stages are detected in somites and smooth muscle cells [8]. Knock-down of *Chap* in the zebrafish resulted in impaired heart looping, cardiac oedema, decreased cardiac contractility, impaired skeletal muscle formation and lethality at 6 days post fertilization [6].

Interestingly, Brugada *et al* were the first to identify the *CHAP/SYNPO2L* locus as a susceptibility locus for atrial fibrillation (AF) in a family with autosomal dominant AF [9]. An independent genome-wide association study (GWAS) in individuals of European ancestry identified a SNP (rs10824026) upstream of CHAP gene as a novel AF locus[10]. More recently, whole-exome sequencing in AF patients revealed that a common variant in CHAP (rs3812629, p.Pro707Leu) was significantly associated with AF [11]. These studies therefore propose the *CHAP* gene as a candidate gene for causing human AF. Although it has been established in animal models that CHAP is essential for cardiac development and function, the role of *CHAP* in disease remains unknown.

Here, we demonstrate that the fetal CHAP isoform (CHAPb) is upregulated in a mouse model of pressure overload-induced hypertrophy. Transgenic expression of fetal isoform CHAPb in the postnatal heart displayed characteristic features of heart disease, such as induction of cardiac hypertrophy, cardiac fibrosis and left atrial enlargement as well as delayed atrio-ventricular conduction, and overall contractile dysfunction. Furthermore, downstream activation of actin signaling in these transgenic mice led to the formation of stress fibers. Together the results indicate that an upregulation of the fetal isoform CHAPb is sufficient to induce cardiomyopathy.

## Methods

### Generation of CHAP Tg mice

Full-length cDNA of mouse CHAPb, preceded by a N-terminal FLAG and Kozak consensus sequence, was fused to the heart-specific murine α-myosin heavy chain (α-MHC) promoter. At the 3’ end of CHAP a poly-A-signal of the human growth hormone (hGH) was included. Plasmid DNA was linearized by digestion with NotI, gel purified and dialyzed against Tris-EDTA (TE) buffer. DNA was injected into pronuclei from mice with a C57BL/6-CBA background. Transgenic (Tg) founder mice were crossed back (5 generations) to C57BL/6 mice to obtain a pure background. For genotyping genomic DNA was isolated from mice tail biopsies and analysed by PCR with a forward primer recognizing the C-terminus of CHAP (5’-TGGTGAAACCCCGTCTCTAC-3’) and a reverse primer recognizing the hGH polyA signal (5’-CAGATTTTCCACTCCTGCAC-3’). All animal experiments were carried out in accordance with Dutch law and approved by the animal experimental committee of the Leiden University Medical Centre (permit number 09129).

Furthermore, the animal experiments were performed conform the guidelines from Directive 2010/63/EU of the European Parliament on the protection of animals used for scientific purposes.

### Western blot

CHAP Tg mice and wild type littermates were sacrificed by cervical dislocation. Hearts were harvested, rinsed in PBS, snap frozen in liquid nitrogen and stored at -80°C until further use. Hearts were homogenized using an ultra-turrax tissue separator (IKA, Germany) in T-PER tissue protein extraction reagent (Pierce) supplemented with: protease inhibitor cocktail tablets (10 μg/ml; Roche, Germany), 0.1 mmol/L dithiothreitol (DTT; Invitrogen) and 1 mmol/L phenylmethanesulfonylfluoride (PMSF; Sigma Aldrich), 5 mmol/L NaF and 1 mmol/L Na_3_VO_4_). Samples were incubated on ice for 15 minutes and centrifuged at 10.000 xg at 4°C for 10 minutes and supernatants were transferred to new tubes. Protein concentration was determined by the Bradford assay (BioRad) using bovine serum albumin as a standard. Proteins (50μg/lane) were separated by SDS-page gel electrophoresis and subsequently blotted using Hybond-P membranes (GE Healthcare) 3 hours at RT. Incubation with the following antibodies was performed overnight at 4°C in 5% milk/TBS-Tween (unless stated else): CHAP (1:200, custom made by Eurogentec MW CHAPa = 140 kDa, MW CHAPb = 110 kDa), actin (1:1000; Millipore BV, MW = 43 kDa), RhoA (1:200, 26C4, Santa Cruz, MW = 24 kDa), alpha-actinin2 (1:800, EA-53, Sigma-Aldrich Chemie, MW = 100 kDa), Ezrin/Moesin/Radixin (1:1000 in 5% BSA/TBS-Tween, Cell Signaling Technology MW moesin = 75 kDa, MW Ezrin and Radixin = 80 kDa), Cofilin (1:1000 in 5% BSA/TBS-Tween, Cell Signaling Technology, MW = 19 kDa), SRF (1:200, G20, Santa Cruz, MW = 40–67 kDa), Myocyte Enhancer Factor 2 (MEF2, 1:200, C21, Santa Cruz, MW = 40–65 kDa) and GAPDH (1:10000, 6C5, Millipore, MW = 38 kDa). Peroxidase-conjugated antibodies used were anti-mouse IgG HRP linked antibody (1:1000, Cell Signaling Technology) and anti-rabbit HRP linked antibody (1:2000, Cell Signaling Technology). For the detection of protein bands SuperSignal West Pico Chemiluminescent Substrate (Pierce) was (the substrate) used.

### Southern blot analysis

Genomic DNA of wild-type and CHAPb Tg mouse tails was extracted in 0.5 ml tail lysisbuffer (50 mM Tris pH 8.0, 100 mM EDTA pH 8.0, 100mM NaCl, 1% SDS and 10 mg/ml prot K) at 55 ^o^C overnight. DNA was precipitated by phenol-chloroform extraction and 10 μg DNA was digested by BamHI (Promega) or XmnI (New England Biolabs) overnight and run on a 1% agarose gel. Probes were generated using the following primers: 5’- AGGGGTCCAGCTCTTTGAAC-3’ and 5’-AGGCTTAAAGCGTCCTCCTC-3’. The PCR products were then radioactively labelled using α-[p32]dATP (PerkinElmer) by random priming (RadPrime, Invitrogen). DNA blots (Hybond-N+, GE Healthcare) were hybridized with the radioactive probe in ExpressHyb Hybridization buffer (Clontech) and visualized by using a Phosphorimager.

### RNA isolation and quantitative PCR

Hearts were homogenized in TRIzol (Invitrogen) using an ultra-turrax tissue separator (IKA, Germany) and RNA was isolated according to the supplier’s protocol. RNA was treated with DNase (DNA-free, Ambion) and cDNA was made with the iScript kit (BioRad). qPCR was performed using the CFX96 Real-Time PCR detection system (Bio-Rad). For details on the primers used see S1 Table. Data were analyzed with Bio-Rad CFX Manager.

### Transmission Electron Microscopy (TEM)

Hearts of CHAPb Tg mice and wild type littermates were collected and left ventricles were used for TEM. For electron microscopy samples were fixed in glutaraldehyde (2.5%) in 0.1 mol/L phosphate buffer for 24 hours, post fixed in 1% OsO_4_ in 0.1 mol/L cacodylate buffer for 1 hour at 4°C, dehydrated in a graded ethanol series and embedded in an epoxy resin. Ultrathin sections were post stained with uranyl acetate and lead citrate and viewed and imaged with a FEI Tecnai 12 transmission electron microscope operated at 120 kV and equipped with an Eagle 4kx4k camera (FEI, Eindhoven, The Netherlands)

### Immunohistochemistry

For histological and immunohistochemical analysis hearts were obtained at different time points (1, 3 and 6 months of age). Mice were sedated by injection of a mixture of 100 μl ketamine, 50 μl rompun and 10 μl atropine in 1ml 0.9% NaCl.

For paraffin sections mice were perfused with 0.9% NaCl and subsequently with 4% paraformaldehyde (PFA). Hearts and other organs (lungs, kidney and liver) were dissected, fixed over night in 4% PFA, dehydrated by ethanol-xylene series and embedded in paraffin. Serial heart sections (5 μm) were made, mounted on starfrost slides (Knittel) and followed by heamatoxilin-eosin (HE) staining, Sirius red staining, and immunohistochemistry as indicated. For all antibody stainings, except Myosin Light Chain 2a (MLC2a), microwave antigen retrieval in citrate buffer (pH 6) was applied. Endogenous peroxidase was blocked by incubating the slides in 0.3% H_2_O_2_ in PBS. Sections were incubated overnight at room temperature with Connexin40 antibody (1:100; Santa Cruz), Connexin43 (1:500; Zymed), MLC2a (1:4000 gift from S. Kubalak) and Troponin I (TnI, 1:800; Santa Cruz). Secondary antibody used was biotin-labeled goat anti-rabbit (Vector Labs) or biotin-labeled horse anti-goat (Vector Labs). Subsequently, the sections were incubated with Vectastain ABC staining kit (Vector Labs) for 45 min. Slides were rinsed in PBS and Tris/Maleate (pH 7.6). 3–3’-diaminobenzidine tetrahydrochloride (DAB, Sigma-Aldrich Chemie) was used as chromogen and Mayer's heamatoxylin as counterstaining. Finally, slides were dehydrated and mounted with Entellan (Merck).

For cryosections mice were perfused with 0.9% NaCl only, then hearts were isolated. Processing of hearts for cryosections was adapted from Bajanca *et al*. [12] Serial sections (5 μm) were made and mounted on starfrost slides (Knittel) and antibody staining was performed as previously described.[13] Antibodies used were CHAP (1:50), myomesin (1:50 E. Elher), α-actinin (Sigma Aldrich), RhoA (1:100, 26C4, Santa Cruz) and pERM (1:50, Cell Signaling Technology). Secondary antibodies used are Cy-3 conjugated anti-mouse (1:250, Jackson Immunoresearch Laboratories) and Alexa488 conjugated anti-rabbit (1:200, Invitrogen). Cell nuclei were counterstained with DAPI (Molecular Probes). Stainings were analyzed with SP5 confocal microscope (Leica).

### Volume measurements

The volume of the atria and ventricle was estimated by the Cavalieri principle of volume estimation. Serial sections were stained for an atrial marker, MLC2a, or cTnI to identify the ventricle. Then a grid was used to estimate the surface of the stained section and the distance between the sections was used to estimate the volume as previously described[14].

### Magnetic Resonance Imaging (MRI)

For MRI measurements 4 wild type and 5 CHAPb transgenic mice of 6 months of age were used. Animals were sedated by 4% isoflurane and MRI images were produced as described below.

All experiments were performed on a vertical 9.4T magnet (Bruker, Ettlingen, Germany) supplied with an actively shielded Micro2.5 gradient system of 1T/m and a 30 mm transmit/receive birdcage RF coil, using Para vision 4.0 software.

MRI protocols

*In vivo*:

At the start of each examination, several 2D FLASH scout images were recorded in the transverse and axial plane of the heart to determine the orientation of the heart.

A modified FLASH sequence with a navigator echo (IntraAngio) was used for retrospective CINE MRI with the following parameters:

Short-axis (oriented roughly perpendicular to the septum) and long-axis (oriented through the apex and aortic valve) cardiac cine MRI images with the navigator positioned through the aortic arch were acquired with the following parameters: hermite-shaped RF pulse 1 ms; FA 10°; 200 avarages, TR 68.1 ms; TE 1.86 ms; reconstruction of 18 cardiac frames; FOV 2.56*2.56 cm2; matrix 192*192; in-plane resolution 133 μm, slice thickness 1.0 mm; total acquisition time approximately 30 min.

Heart function was assessed using MASS for Mice 5.1 software package (Leiden, The Netherlands) by manual delineation of LV and RV borders.

Based on these segmented areas, LV and RV area, end-diastolic volume (EDV), end-systolic volume (ESV), LV stroke volume (SV), and LV ejection fraction were computed automatically.

### Electrocardiogram (ECG) measurements

ECG measurements on wild type and CHAPb Tg mice of 6 months of age were performed as described in Henkens *et al*. [15]. In brief, before ECG recording, animals were anesthetized by inhalation of 4% isoflurane. Anesthesia was maintained at 2% isoflurane administration and ECG measurements were recorded for at least one minute. The data were analysed with the program LEADS and heart rate, PR duration and P duration were calculated.

### Sarcomere measurements

Hearts of CHAPb Tg mice and wild type litter mates were isolated at different time points (1 month and 3 months) and snap frozen in liquid nitrogen. Single cardiomyocytes were obtained by grinding hearts in liquid nitrogen. Cardiomyocytes were defrosted in relaxing solution (pH 7.0; 1 mmol/L free Mg^2+^ 1, 145 mmol/L KCl, 2 mmol/L EGTA, 4 mmol/L ATP, 10 mmol/L imidazole). Then cardiomyocytes were treated with relaxing solution containing 1% Triton-x-100 for 5 minutes, to remove the membranes. Cardiomyocytes were washed twice in relaxing solution to remove Triton-X-100. Single cardiomyocytes were placed between a force transducer and piezoelectric motor. Isometric force measurements were performed at 15°C and a sarcomere length, measured in relaxing solution, of 2.2 μm. The calcium concentrations of relaxing and activation solution (pH 7.1) were 10^−3^ and 30 μmol/L, respectively. Solutions with intermediate free [Ca^2+^] were obtained mixing relaxing and activation solution. The first control activation was performed at maximum [Ca^2+^] and thereafter, the resting sarcomere length was set to 2.2 μm again. The second control measurement was performed to calculate the maximum isometric tension (force divided by cross-sectional area). The next measurements (4–5) were performed in submaximal [Ca^2+^], followed by a control measurement. Force values obtained from submaximal [Ca^2+^] were normalized to control values.

### Protein phosphorylation

Protein phosphorylation of sarcomeric proteins was measured in hearts of CHAPb Tg and wild type litter mates at 3 months of age. Briefly, samples were separated by SDS-PAGE and gels were stained by SYPRO Ruby stain or Pro-Q Diamond Phosphoprotein Stain. A detailed description of the procedure can be found in Zaremba *et al*. [16].

### Statistical analysis

**Fig 1:**
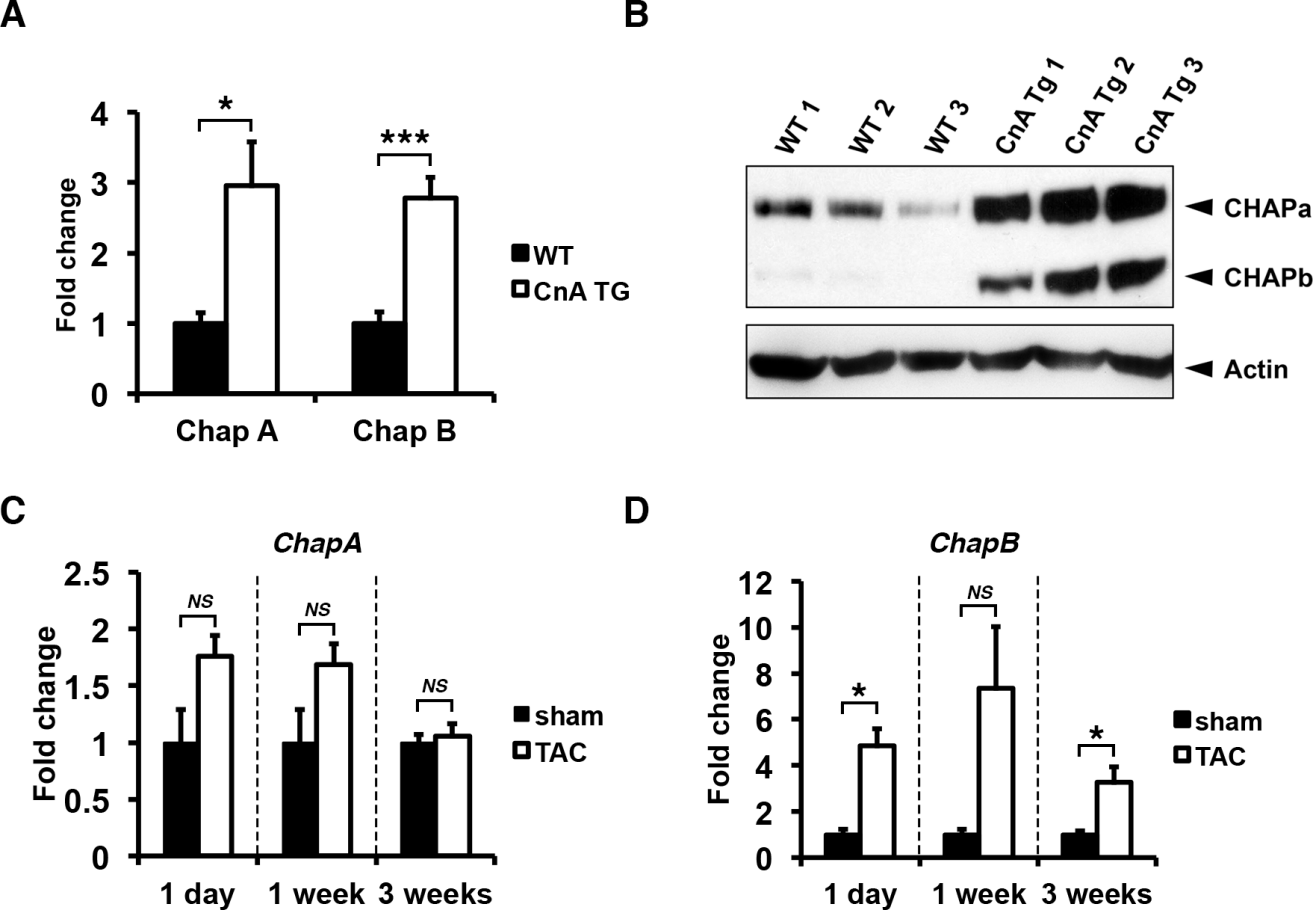
Chap is upregulated in mouse models of pathological cardiac hypertrophy. (A) Quantitative RT-PCR showing increased mRNA levels of both ChapA and B in hearts of transgenic mice expressing constitutively active calcineurin A (CnA Tg) (WT: n = 5, CnA Tg: n = 6; Students *t-test*: CnA Tg vs WT). (B) Western blot for CHAPa and CHAPb in adult wildtype hearts CnA Tg hearts (WT: n = 5, CnA Tg: n = 6). In wildtype hearts, CHAPa is the dominant isoform while in CnA Tg hearts both CHAPa and b were significantly upregulated. (C) Quantitative RT-PCR for ChapA showing mice subjected to transverse aortic constriction (TAC) for 1 day, 1 week and 3 weeks (n = 4, n = 4, and n = 10 respectively), with no significant regulation of ChapA mRNA compared to sham controls (n = 3, n = 3 and n = 7 respectively). (D) Quantitative RT-PCR for ChapB showing mice subjected to TAC with significant upregulation of ChapB mRNA (number of mice are same as in C). Gapdh was used as internal control in all experiments. *T-test* with Welch’s correction TAC vs sham: *, p<0.05; **, p<0.01;***, p<0.001.

Data were analysed with GraphPad Prism. All data are expressed as mean + the standard error of the mean (SEM). Statistical analysis between two groups was performed using Student’s unpaired *t-test*, or *t-test* with Welch’s correction where appropriate (Fig 1C and 1D). All analyses were 2-tailed and a *P* value less than 0.05 was considered statistically significant.

## Results

### CHAPb is upregulated in cardiac hypertrophy

Many genes expressed during heart development are reactivated during heart failure. Since we previously identified an important role for CHAP during cardiac development we investigated both isoforms of CHAP (S1 Fig) in an *in vivo* model of experimental heart failure.

Transgenic mice with cardiac-specific overexpression of a constitutively active form of calcineurin display a massive cardiac hypertrophic response [3]. In hearts of adult calcineurin Tg mice, expression of both isoforms was increased compared to expression in wild type (wt) mice at RNA (Fig 1A) and protein level (Fig 1B). Next we investigated the expression of cardiac *Chap* in a model of pressure overload-induced hypertrophy, transverse aortic constriction (TAC). Expression of *ChapA* was not significantly changed at different times after TAC (Fig 1C), whereas *ChapB* was significantly increased as soon as 1 day of TAC and was maintained until three weeks of TAC, as compared to sham controls (Fig 1D). In summary, experimental models of cardiac hypertrophy demonstrated increased levels of both CHAP isoforms, although reactivation of fetal isoform CHAPb was predominant in the pressure overload model.

### Ectopic CHAP expression in transgenic mice

In order to investigate a possible role for CHAPb in the development of cardiac hypertrophy, we generated cardiac-specific CHAPb Tg mice. These Tg mice express a N-terminal FLAG-tagged CHAPb isoform in the postnatal heart under control of the cardiac-specific α-myosin heavy chain (MHC) promoter (S2A Fig). Injection into C57BL/6-CBA zygotes resulted in three CHAPbTg founders among 30 animals, that showed different copy number levels as determined by Southern blot (S2B Fig). Analysis of hearts of the Tg line with intermediate copy numbers by qPCR demonstrated upregulation of *ChapB* compared to wt mice (S2C Fig). Western blot analysis of Tg hearts showed a strong increase in CHAPb protein levels (110 kDa), when compared to wt hearts. This was confirmed by the presence of anti-FLAG immunoreactivity in the Tg hearts only (S2D Fig).

### CHAPb expression in adult mouse heart leads to enlarged atria

Southern blot analysis of founders showed high CHAPb copy number in transgenic line 29 and intermediate CHAPb copy number in line 14 compared to endogenous genomic levels of CHAP (S2B Fig). The highest CHAPb expressing founder (Tg line 29) died after 7 months, without progeny. Progeny of the Tg line 14 died spontaneously at approximately 6 months. Hearts of both transgenic lines displayed comparable phenotypes with enlargement of left and right atrium (S3A and S3B Fig). Interestingly, all mice that spontaneously died displayed severely enlarged left atria for both transgenic lines. Histological analysis revealed that the left atrium was filled with a chronic and organized thrombus. Furthermore, thickness of the septum, left and right ventricle wall was increased, suggesting cardiac hypertrophy. This was confirmed by hypertrophy of cardiomyocytes at higher magnifications (S3C Fig).

### Expression of fetal isoform CHAPb in adult hearts leads to cardiac hypertrophy

**Fig 2:**
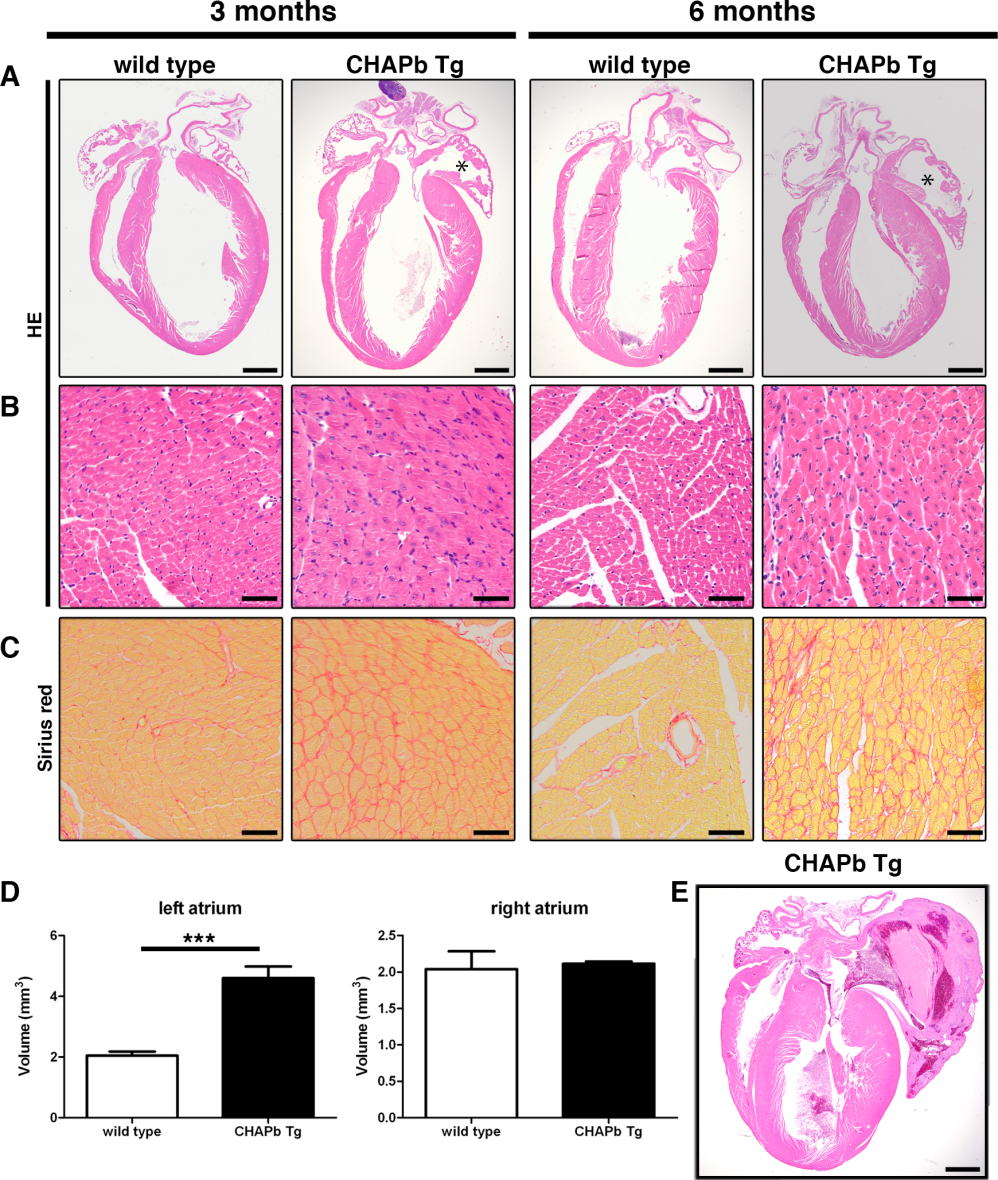
Hypertrophy and left atrial enlargement in CHAP Tg hearts. (A-C) Wt (left panels) and CHAPb Tg (right panels) at 3 and 6 months of age. (A) HE stained overview section. The left atrium in CHAPb Tg hearts is enlarged (indicated by *) compared to wt litter mates. (B) Higher magnification of left ventricle. In the left ventricle of CHAPb Tg hearts the cardiomyocytes are hypertrophic. (C) Sirius red staining of the left ventricle showing increase in interstitial fibrosis in CHAPb Tg. (D) Myocardial volume of the left atrium (left panels) and right atrium (right panels) in wt (white bars) and CHAPb Tg (black bars) hearts at 3 months of age (wt n = 4, Tg n = 4, T-test p< 0.001). (E) CHAPb Tg heart with severe phenotype showing pronounced atrial enlargement, filled by a thrombus and thickening of the ventricles. Scale bars 1 mm in A and D, 50 μm in B, C.

We analyzed CHAPbTg mice at one, three and six months of age. At one month, hearts of wt and Tg mice were indistinguishable (S4A–S4C Fig). At three months the left atrium of Tg hearts was enlarged without signs of thrombus formation (Fig 2A). Tg hearts showed obvious cardiomyocyte hypertrophy with enlarged nuclei (Fig 2B) and interstitial fibrosis, indicated by Sirius Red staining (Fig 2C). To determine the extent of cardiac hypertrophy, we measured atrial and ventricular volume at these ages. Myosin Light Chain 2a (MLC2a) and Troponin I (TnI) staining were used to identify the atria and ventricle, respectively. As expected, the volumes of the atria at one month was similar in wt and Tg mice (S4D Fig) but at three months, the left atrial volume of Tg mice was significantly greater than in wt (wt: 2.046 mm^3^ ± 0.1346 n = 4, Tg: 4.598 mm^3^ ± 0.3853 n = 4, p< 0.01; Fig 2D) whereas the volumes of the right atria were similar (wt 2.041 mm^3^ ± 0.2431 n = 4, Tg 2.114 mm^3^ ± 0.03001 n = 4, p = NS). Furthermore, despite the hypertrophy evident in individual cardiomyocytes of the left ventricle, the overall ventricular wall volume was not different in Tg mice (wt 57.81 mm^3^ ± 6.352 n = 4, CHAPbTg 51.31 mm^3^ ± 2.705 n = 4, p = NS).

By six months, left atrial enlargement was consistently more pronounced in Tg mice and was associated with cardiomyocyte hypertrophy and interstitial fibrosis (Fig 2B and 2C, S5 Fig). Occasionally, intraventricular thrombi were identified (Fig 2E). In general, changes in Tg hearts were more pronounced at six months, displaying features that resemble cardiomyopathy, including sudden death.

### Activation of hypertrophic gene program and fibrosis in CHAPb Tg hearts

**Fig 3:**
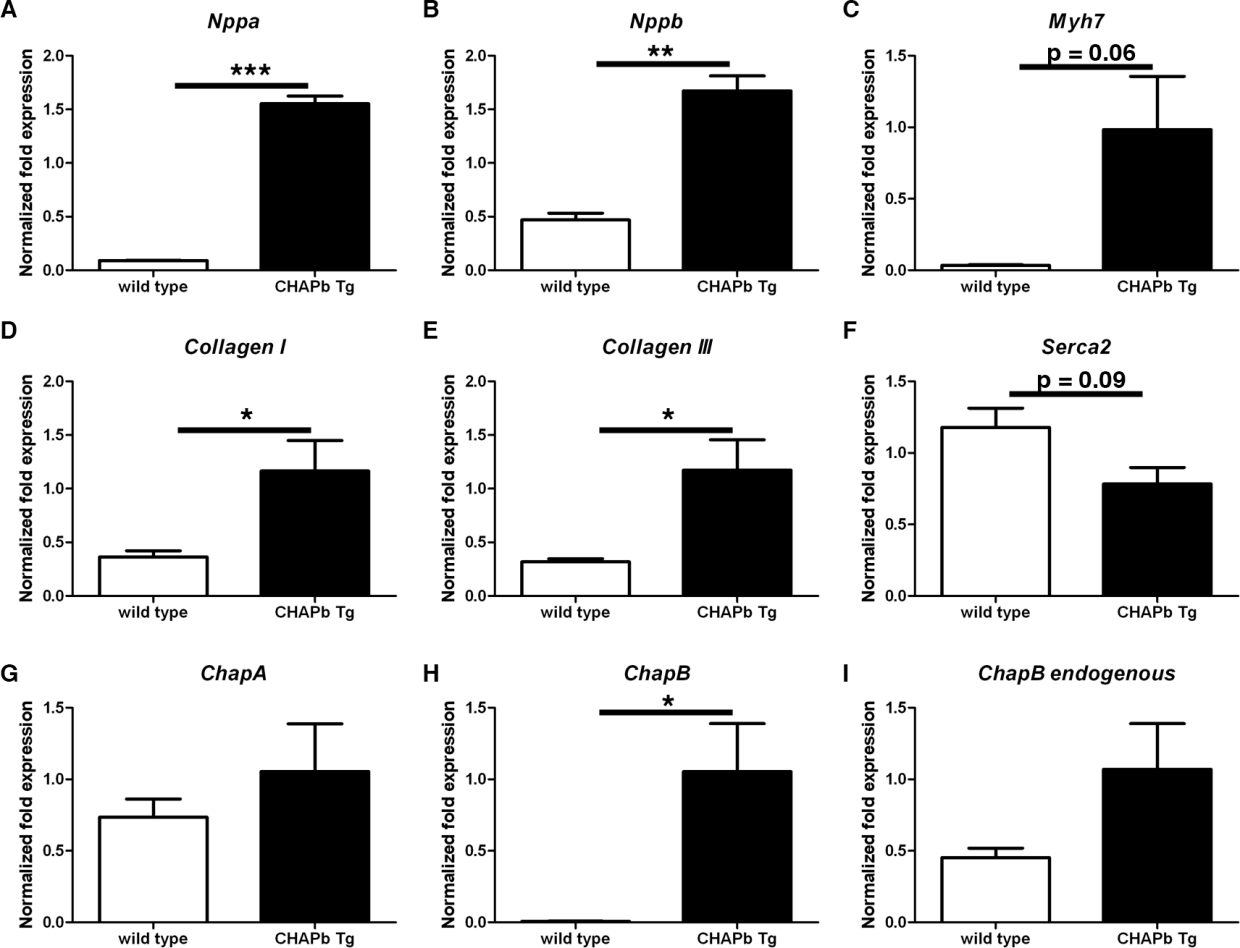
Expression of hypertrophy markers and collagens in left ventricle. qPCR analysis showing mRNA expression of *Nppa* (A), *Nppb* (B), *Myh7* (C), *CollagenI* (D) *CollagenIII* (E), *Serca2* (F), *ChapA* (G), *ChapB* (H) and endogenous *ChapB* (I) in the left ventricle of wt (white bars) and CHAPb Tg (black bars) mice. *Gapdh*, *Pgk* and *H2a* were used as internal controls. (wt n = 3, Tg n = 3, T-test: *, p<0.05; **,p<0.01;***, p<0.001).

We isolated RNA from left ventricles of six-month-old wt (n = 3) and Tg (n = 3) mice to investigate gene expression. A hallmark of hypertrophy is re-expression of fetal cardiac genes, such as atrial natriuretic factor (ANF, *Nppa*), brain natriuretic peptide (BNP, *Nppb*) and β-MHC (*Myh7*). qPCR analysis showed significant upregulation of *Nppa* (17.2x, p<0.01, Fig 3A), *Nppb* (3.6x, p<0.01, Fig 3B) and *Myh7* (28.3x, p = 0.0634, Fig 3C) in Tg hearts. These findings corroborate the hypertrophic response observed morphologically.

Fibrosis is characterized by increased collagen production. To confirm Sirius red evidence, we analyzed the expression of *Collagen I* and *III*, the major fibrin forming collagen types. Increased expression of *Collagen I* (3.2x, p = 0.0506, Fig 3D) and *III* (3.7x, p = 0.0404, Fig 3E) was indeed observed in Tg animals, as expected. Moreover, mRNA expression of *Serca2*, which encodes a protein involved in Ca^2+^ cycling [17] and is generally down-regulated in cardiac hypertrophy, was decreased in Tg hearts (Fig 3F).

Finally, we examined endogenous *Chap* isoforms in Tg mice. *ChapA* mRNA expression was similar in wt and CHAPb Tg hearts (Fig 3G) and although endogenous *ChapB* appeared slightly increased, this was not statistically significant (Fig 3I). Exogenous *ChapB* (Fig 3H) was, as expected, strongly upregulated in Tg hearts compared to wt.

### Expression of ChapB in adult heart leads to loss of Connexins in the left atrium and conduction disturbances

**Fig 4:**
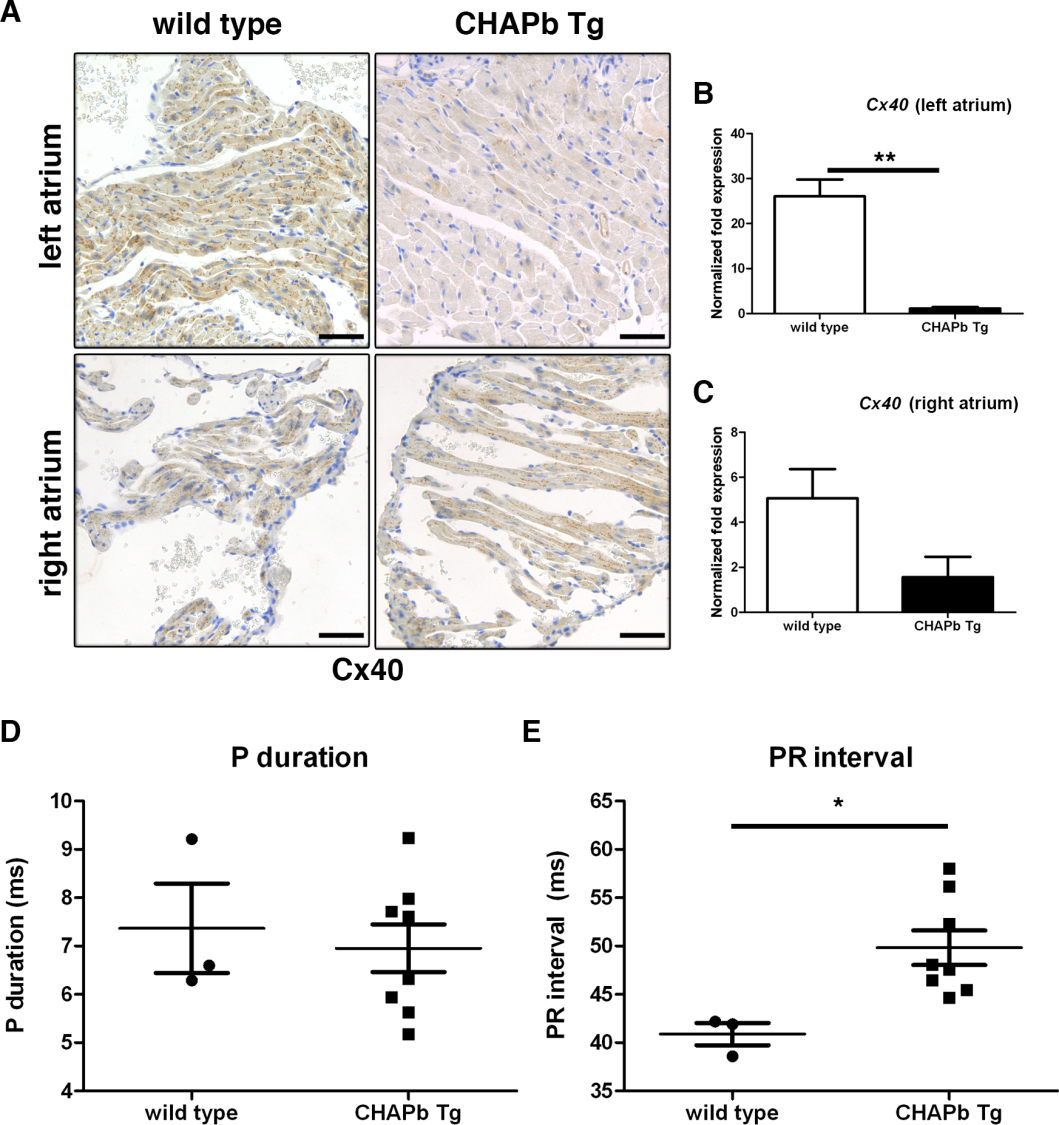
Decreased Connexin 40 expression in CHAPb Tg left atrium at 6 months of age leads to atrio-ventricular conduction delay. (A) Immunohistochemical staining showing Connexin 40 expression in wt (left panels) and CHAPb Tg (right panels) left (upper panels) and right (lower panels) atria. (B,C) qPCR analysis of Connexin 40 expression in the left (B) and right (C) atrium (wt n = 3, Tg n = 3 T-test, **,p<0.01). (D,E) ECG analysis of wt (n = 3) and CHAPb Tg (n = 8) mice showing P duration (N.S.) (D) and PR interval (p = 0.02, T-test) (E). Scale bars 50 μm.

Since cardiomyopathies are associated with disturbed electrical conductance and cardiac arrhythmia, we investigated the most predominant Connexin (Cx) isoforms in atria and ventricles. Cx40 is the main isoform expressed in atria. Cx43 is expressed at higher levels in ventricles and at lower levels in the atria [18]. At one month, there was no difference in Cx40 and Cx43 expression between wt and Tg (S6A and S6B Fig). At three months, however, enlargement of the left atrium was accompanied by loss of expression of both Cx40 and Cx43, although expression in the right atrium (S6C and S6D Fig) and ventricles were unchanged. This decrease in Cx40 levels in the left atrium was more pronounced at six months (Fig 4A). These findings were confirmed by qPCR, which showed that Cx40 (-23x, p<0.01, Fig 4B) and 43 (-6.9x, p<0.01, S5E Fig) were both downregulated in the left atrium. Similar to the protein data, mRNA expression of Cx40 and Cx43 in the right atrium was unchanged (Fig 4C and S6F Fig).

To investigate whether the loss of expression of Cx40 and 43 was correlated with conduction disturbances, we performed ECG measurements of wt and Tg mice. Heart rate was similar between wild type and Tg mice (S6G Fig) whereas the PR interval was increased in Tg mice (wt 40,89 ms ± 1,150 n = 3, Tg 49,83 ± 1,784 n = 8, p<0.05; Fig 4E) indicating a conduction delay from atria to ventricles. No signs of atrial fibrillation were observed, as the duration of the P-top was unchanged (wt 7,367 ms ± 0,9260 n = 3, Tg 6,955 ms ± 0,4934 n = 8, p = ns; Fig 4D) [19].

### CHAPb Tg mice show decreased cardiac performance

**Fig 5:**
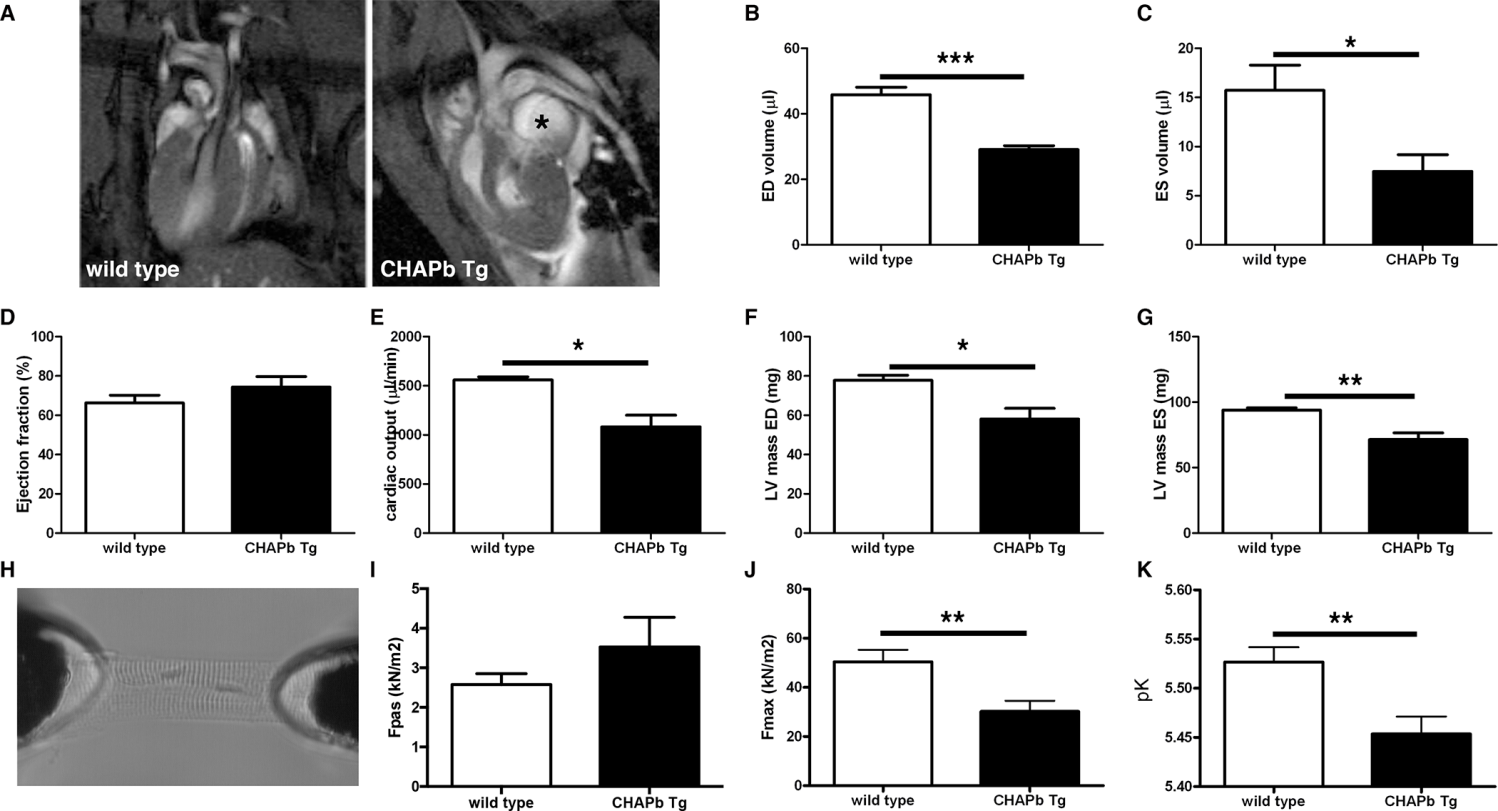
CHAPb Tg mice show decrease cardiac performance. **(**A) Representative 4-chamber view MRI images of wt and CHAPb Tg heart. Enlarged LA in CHAPb Tg is indicated with *. (B-G) MRI measurements of the left ventricle of wt (n = 4, white bars) and CHAPb Tg (n = 5, black bars) animals at 6 months of age. ED volume (B), ES volume (C), ejection fraction (D), cardiac output (E), LV mass ED (F) and LV mass ES (G). (H) Experimental setup for single membrane-permeabilized cardiomyocyte measurements to determine sarcomere function. Sarcomere force measurements at one month of age showed no difference in passive force (I; Fpas), while maximum force (J; Fmax) and Ca^2+^-sensitivity (K; pCa_50_) were reduced in CHAPb Tg (n = 11, black bars) compared to wt (n = 12, white bars). ED (end diastolic), ES (end systolic) and LV (left ventricular). *t*-test: *, p<0.05; **, p<0.01; ***, p<0.001.

To determine whether the phenotypic changes in Tg hearts affected cardiac performance, we performed MRI measurements on 4 wt and 5 Tg male mice at six months. Enlargement of the left atrium was clearly visible in the scans. We then calculated the functional parameters of the left and right ventricle. The results of the left ventricle are shown in Fig 5. Representative scans for wt and Tg are shown in Fig 5A. Both end diastolic volume (wt 45.81 μl ± 2.297 n = 4, Tg 29.08 μl ± 1.176 n = 5, p<0.01; Fig 5B) and end systolic volume (wt 15.72 μl ± 2.570 n = 4, Tg 7.474 μl ± 1.702 n = 5, p<0.05; Fig 5C) were significantly decreased, resulting in a similar ejection fraction in wt and Tg mice (wt 66.26% ± 3.972 n = 4, Tg 74.35% ± 5.376 n = 5, p = ns; Fig 5D). However, the cardiac output was significantly decreased in Tg mice compared to wt (wt 1559 μl/min ± 32.20 n = 4, Tg 1083 μl/min ± 117.6 n = 5, p<0.01; Fig 5E). Both the left ventricular mass of end diastole (wt 77.79 mg ± 2.572 n = 4, Tg 58.11 mg ± 5.399 n = 5, p<0.05; Fig 5F) and end systole (wt 93.97 ± 1.784 n = 4, Tg 71.39 ± 5.127 n = 5, p<0.01; Fig 5G) was decreased in Tg mice. Similar data were obtained for the right ventricle (S7 Fig). Overall, these data show that CHAPb overexpression impairs diastolic and systolic function in both the left and right ventricle.

### CHAPb Tg mice display decreased force generating capacity of cardiac sarcomeres

To investigate the functional properties of cardiac sarcomeres, we measured force development at different calcium concentration in membrane-permeabilized single cardiomyocytes (Fig 5H) from wt and Tg sarcomeres at one month. We observed no difference in passive force (Fig 5I) whereas at the highest calcium concentration (30 μmol/L) force development was decreased in Tg cardiomyocytes (wt 50.40 kN/m^2^ ± 4.911 n = 12, Tg 30.23 kN/m^2^ ± 4.346 n = 11, p = 0,006; Fig 5J). Furthermore, sarcomere Ca^2+^-sensitivity (pCa_50_, the calcium concentration at which half of the maximum force was generated) was also decreased in Tg sarcomeres compared to wt (wt 5.53 ± 0.02 n = 12, CHAPb Tg 5.45 ± 0.02 n = 11, p = 0,0046; Fig 5K). The reduction in both maximal force and pCa_50_ shows that Tg sarcomeres have less force generating capacity compared to wt sarcomeres. The phosphorylation status of the sarcomeric proteins troponin I and T, myosin binding protein C and myosin light chain 2, remained unchanged (S8 Fig). These results correlated with those from MRI, where we found decreased diastolic function, indicating a stiffer heart.

### Sarcomeric organization is disturbed in CHAPb Tg hearts

**Fig 6:**
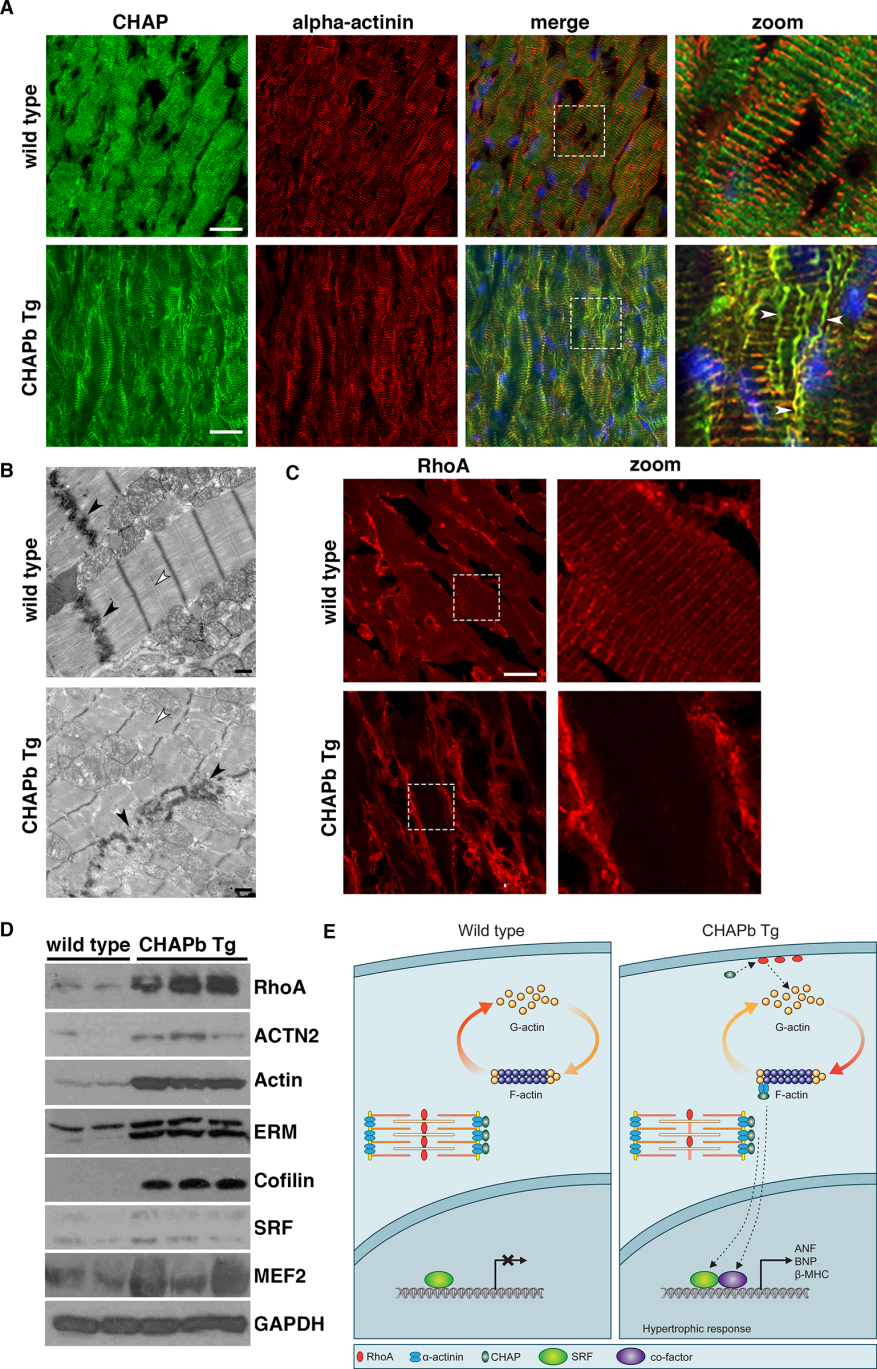
Sarcomeric organization is disturbed and actin signalling is increased in CHAPb Tg hearts. (A) Immunostaining for CHAP (green) and α-actinin (red) show stress fiber formation (white arrows) in CHAP Tg hearts (B) Electron microscopy analysis of wt and CHAPb Tg hearts at 6 months of age. In CHAPb Tg the sarcomeres were irregular and Z-discs and intercalated discs (black arrow heads) are disorganized, while M-bands are absent (white arrow heads) (C) Wt and CHAPb Tg hearts at 6 months of age stained for RhoA (red). In wt mice RhoA is localized at the membrane of cardiomyocytes and shows a sarcomeric expression pattern. In CHAPb Tg hearts sarcomeric expression of RhoA is absent and membrane expression is increased. (D) Western blot analysis of 2 wt and 3 CHAPb Tg hearts at 6 months of age for RhoA (24kDa), α-actinin (100kDa), actin (42kDa), Ezrin(80 kDa)/moesin(80 kDa)/radixin (75 kDa; ERM), cofilin (19kDa), SRF (40 – 67kDa) and MEF2 (40-65kDa). GAPDH (38kDa) is used as loading control. (E) Working model: in adult wt mice CHAPa is localized at the Z-disc, leading to abundance of monomeric G-actin and subsequent low expression of SRF target genes. In CHAPb Tg mice CHAPb expression results in activation of RhoA, leading to a shift from G-actin to F-actin, binding of co-factors to SRF and activation of SRF target genes, such as ANF, BNP and β-MHC. Scale bars 20 μm in A and C, 1 μm in B.

Next we investigated the sarcomeric organization in cryosections of wt and Tg hearts. Immunohistochemical staining showed that CHAP and α-actinin-2 were co-localized at the Z-disc of wt and Tg cardiomyocytes (Fig 6A), whereas CHAP did not overlap with myomesin (M-band protein; S9 Fig). However, in the Tg hearts, CHAP was also localized in fibers, which appeared to be perpendicular to the sarcomeres and resembled the formation of stress fibers. These fibers stained with α-actinin (Fig 6A), but not myomesin (S9 Fig). These stress fibers were already visible at one month. To study the sarcomeres in more detail we performed Transmission Electron Microscopy on 6 months old wt and Tg hearts. Organization of wt sarcomeres was regular with well-formed Z-discs, M-bands and intercalated discs, whereas Tg sarcomeres were irregular and both Z-disc and intercalated discs were disorganized (Fig 6B). Interestingly, the M-Band was not clearly visible in sarcomeres of CHAPb Tg mice.

### Activation of the actin signalling pathway in CHAPb Tg hearts

Since synaptopodin, a family member of CHAP, has been associated with actin signalling in brain and kidney and since this pathway is involved in cardiac hypertrophy, we investigated the actin signalling pathway in Tg hearts. Staining of GTPase RhoA, a key component of the actin signalling pathway, in hearts of six months showed sarcomeric expression pattern in wt, while in Tg expression was increased and, interestingly, was displaced from its sarcomeric localization (Fig 6C). To further confirm the CHAPb-dependent activation of actin signalling, we examined downstream effectors. Indeed, phosphorylation of Ezrin/radixin/moesin (ERM), a family of proteins involved in Rho-dependent signalling and linking the actin cytoskeleton to the plasma membrane [20, 21], was increased in Tg hearts and displayed a similar localization as CHAP (S10 Fig). Increased protein expression levels of RhoA, actin, α-actinin, ERM, cofilin, and downstream actin-dependent transcription factors serum response factor (SRF) and myocyte enhancer factor-2 (MEF2) in hearts of Tg compared to wt (Fig 6D) confirmed these findings. These results suggested CHAPb dependent activation of the actin-signalling pathway, from membrane to the nucleus, which may contribute to the molecular, phenotypic and functional alterations observed in the Tg mice.

## Discussion

Here we show that the Z-disc protein CHAP is upregulated in experimental models of cardiac hypertrophy and that cardiac-specific overexpression of the fetal isoform CHAPb is sufficient to induce various aspects of heart remodelling and decreased heart function. CHAP was initially identified as a gene up-regulated during the differentiation of human embryonic stem cell-derived to cardiomyocytes [7]. In mice and human two CHAP isoforms were identified which differ in their temporal expression patterns: the shorter isoform CHAPb is expressed during embryonic development in the heart, somites and muscle precursors, whilst CHAPa is expressed in the adult heart and skeletal muscle. Morpholino knockdown of *Chap* in zebrafish showed that it is essential for heart development. Furthermore, overexpression of “embryonic” CHAPb, but not “adult” CHAPa, in rat cardiomyocytes led to dissociation of α-actinin-2 from the Z-disc [6, 22, 23]. Here we show that CHAPb Tg mice displayed features which are comparable to cardiomyopathy, such as cardiac hypertrophy, interstitial fibrosis, diastolic dysfunction, and disturbed electrical conductance, which led to higher mortality.

### CHAPb induces cardiac hypertrophy, left atrial enlargement and fibrosis

CHAPb overexpression in Tg hearts was apparent both in qPCR and Western blot analysis. This had no effect at one month but by three months, molecular markers of hypertrophy were upregulated and cardiomyocyte hypertrophy without ventricular wall thickening, left atrial enlargement and interstitial fibrosis were clearly evident. This was more pronounced at six months and occasionally accompanied by wall thickening of the left and right ventricles and septum. In agreement with increasing severity of the phenotype, Tg mice died spontaneously from six months of age. Occasionally thrombi were found in the left atria, which may have disturbed blood flow in the left atrium and impaired ventricular filling. Reduced ventricular filling may eventually contribute to pulmonary venous congestion and development of pulmonary oedema. Although we did not observe pulmonary oedema overall (indicated by preserved lung weights) in Tg mice, we cannot exclude that this occurred in individual mice. Indeed, occasionally MRI, revealed white/grey areas in lungs of Tg mice, suggesting fluid retention.

### Impaired structural and electrical organization at intercalated discs in CHAPb Tg hearts

In addition to the morphological and histological changes in Tg heart, we observed changes at the intercalated discs of cardiomyocytes. In particular, gap junction proteins Cx40 and Cx43 were markedly downregulated in the left atrium of Tg hearts, which was already visible at three months but more pronounced at six months of age. Since gap junctions are crucial for fast spreading of action potentials between cardiomyocytes, impaired expression of gap junctions expected to affect electrical guidance. Indeed, decreased connexins expression in the left atrium correlated with conduction disturbances, evidenced by increased PR interval, the time required for conduction between atria and ventricles. Loss of expression of Cx40 and 43 has been associated previously with conduction disturbances in mice. In Cx40^-/-^ mutant mice, various conduction disturbances have been reported, including increased PR interval [22, 23]. In Cx43^+/-^ mice ventricular conduction was delayed,[24] although no change in atrial conduction was observed [25]. In other hypertrophy mouse models, down-regulation of Cx’s in the atria also correlated with conduction disturbances [26–28].

Although decreased expression of connexins and impaired electrical guidance in Tg mice was limited to the atria, detailed analysis of individual cardiomyocytes by electronmicroscopy showed that there was disorganization of Z-discs and intercalated discs throughout the whole heart. Sarcomeric disorganization would suggest impaired contractility and/or relaxation of cardiac muscle, affecting cardiac function.

### CHAPb Tg mice display cardiac diastolic dysfunction

MRI measurements indicated that Tg mice had normal left ventricular ejection fraction. Similarly, in patients with heart failure, almost half (47%) still have a normal ejection fraction [29]. Ejection fraction is determined by differences in both end-diastolic and end-systolic volumes and if both parameters are changed, as occurs in Tg mice, ejection fraction may be unaffected. The decreased diastolic volumes observed in Tg mice may suggest impaired relaxation during diastole (diastolic dysfunction), which relates to impaired Ca^2+^ cycling in cardiomyocytes. Contraction of cardiomyocytes is achieved by entry of Ca^2+^ through L-type channels, which causes Ca^2+^ release into the cytosol, immediately followed by Ca^2+^ release from the sarcoplasmic reticulum (SR). Subsequently, Ca^2+^ binds to sarcomeric protein troponin C to initiate contraction. On the other hand, relaxation occurs through uptake of the Ca^2+^ into the SR via SERCA2. In Tg cardiomyocytes, re-uptake of Ca^2+^ might be affected, although reduced *SERCA2* mRNA levels in Tg mice did not reach statistical significance [17, 30]. In addition, measurements in membrane-permeabilized cardiomyocytes revealed reduced force-generating capacity of Tg sarcomeres, which may in part underlie the significant reduction in cardiac output observed in Tg compared to wt mice. In primary cardiomyopathy, alterations in Ca^2+^-sensitivity have been reported, which may depend on the location of the mutation in affected genes. Perturbations in Ca^2+^-sensitivity of the sarcomeres may result in development of HCM or DCM [31]. As overexpression of CHAPb reduced Ca^2+^-sensitivity, it would be interesting to investigate the role of CHAP in primary cardiomyopathy cases.

### Activated actin signalling in CHAPb Tg hearts

We found that expression of RhoA is increased in Tg hearts. RhoA belongs to the family of the small GTPases. Its effects on the actin cytoskeleton are mediated through stimulation of Rho Kinase (ROCK) [32]. RhoA is expressed in the heart during embryonic development, is down-regulated after birth and re-expressed in cardiac hypertrophy [33]. Furthermore, RhoA Tg mice develop a phenotype that resembles DCM, which is accompanied with conduction disturbances [34]. Alternatively, inhibition of ROCK reduces pressure overload-induced hypertrophy in rats [35]. It has been previously shown that synaptopodin, is involved in actin stress fiber formation by preventing proteasomal degradation of RhoA [36]. Like synaptopodin, CHAPb might be involved in the stabilization of RhoA, which may lead to actin stress fiber formation and induction of hypertrophy via the Rho-ROCK pathway.

### CHAP is involved in the SRF/MEF-2 actin signalling pathway

We observed increased expression of transcription factors SRF and MEF-2 in Tg hearts. Previously, it has been demonstrated that Striated muscle activator of Rho signalling (STARS), an actin-bundling protein localized at the Z-disc, is associated with both SRF and MEF-2 signalling. STARS is induced by MEF-2, which in turn regulates the formation of F-actin fibers via RhoA, leading to depletion of the monomeric globular actin (G-actin) pool. Consequently, a reduction of G-actin may lead to activation of SRF mediated transcription [37–39]. Previous studies in mice have indicated the relevance of SRF in the development of cardiomyopathy [40, 41]. In addition, the importance of SRF in cardiomyocyte function, maintenance and regulation was shown in experiments in which it was knocked-down: expression of cytoskeletal genes, such as α- and β-MHC, cardiac α-actin and smooth muscle actin were decreased and also, interestingly, CHAP [42, 43]. With respect to the MEF2 transcription factor family (MEF-2a, c and d), Tg mice overexpressing these genes in the heart also showed cardiac hypertrophy and stress-dependent cardiac remodelling [44–46]. The phenotypes of the different Tg models do not overlap completely with the phenotype observed here, suggesting that additional pathways may be involved in the CHAPb induced hypertrophy. In summary, our study suggests a model in which increased levels of CHAPb may activate actin signalling leading to subsequent activation of cardiac transcription factors MEF2 and SRF, initiating a hypertrophic response and structural and functional changes in cardiomyocytes (Fig 6E).

### Conclusion

We show that postnatal re-expression of the embryonic isoform of CHAP, CHAPb, is sufficient to cause cardiomyopathy with diastolic cardiac dysfunction and conduction disturbances, which is associated with sarcomere dysfunction and activation of actin signalling and of the downstream transcription factors MEF-2 and SRF. Recently, Kong *et al* [4] demonstrated that apart from alterations in gene expression, changes in alternative mRNAs of sarcomeric genes particularly are associated with heart failure^.^ These findings and our study demonstrate the importance of correct dosage of sarcomeric gene isoforms and substantiate further investigation in developmentally (dys-) regulated alternative isoforms of other cardiac gene products.

Recently the genomic locus of CHAP (*SYNPO2L*) has been associated with AF and blood pressure regulation [9–11, 47], which suggests that CHAP is a clinically relevant gene. The functional role of CHAP in AF and regulation of blood pressure remains elusive. It is therefore important to perform in depth functional studies of CHAP *in vivo* to gain insight in the molecular mechanism of disease.

Taken together, our results identify CHAPb as a novel component in the pathology of cardiomyopathy, a potential therapeutic target, and new candidate gene for screening mutations in familial cardiomyopathies and atrial fibrillation.

## Supporting information

S1 Fig Genomic organization and isoforms of mus musculus *Chap*. (A) Genomic organization of the mouse *Chap* gene on chromosome 14 (B) mRNA isoforms of CHAP resulting from differential usage of transcriptional start sites (C) CHAPa and CHAPb protein isoforms.
(JPG)

S2 Fig Generation and validation of Chap B TG mouse. (A) Schematic overview showing the construct used to generate transgenic mice. FLAG-CHAPb cDNA is downstream of the α-MHC promoter and upstream a human Growth Hormone polyA signal. (B) Southern blot analysis of wild type and CHAPb Tg genomic DNA showing intermediate copy number in line 14 and high copy number in line 29, compared to wild type copy numbers. Hearts of CHAPb founder line 29 (left panels) and line 14 (right panels). (C) qPCR analysis of *ChapB* in wt (white bars, n = 6) and CHAPb Tg hearts (black bars, n = 3). *Gapdh* was used as internal control (D) Western blot showing CHAP and FLAG expression in wt (n = 2) and CHAPb Tg (n = 3) hearts. GAPDH was used as loading control.
(JPG)

S3 Fig Hearts of CHAPb Tg founders that died spontaneously. (A) Hearts showing enlarged atria and malformed ventricles. (B) HE stained overview section showing enlarged atria and thickened ventricles. (C) Higher magnification of the left ventricle. Scale bars in B 1 mm, in C 50 μm.
(JPG)

S4 Fig HE and Sirius red staining of CHAPb Tg hearts at one month of age. Wt (left panels) and CHAPb Tg (right panels) at 1 month of age (A-C). A) HE stained overview section. (B) Higher magnification of left ventricle. (C) Sirius red staining of the left ventricle. (D-E) Volume of the left atrium (D) and right atrium (E) in wt (n = 4, white bars) and CHAPb Tg (n = 4, black bars) hearts (*t*-test, NS). Scale bars 1 mm in A, 50 μm in B, C.
(JPG)

S5 Fig Interstitial fibrosis in CHAPb Tg left atrium at six months of age. Sirius red staining on atrial sections of wild type (left panel) and CHAPb Tg mice shows increased interstitial fibrosis and increased myocytes size in CHAPb Tg atria.
(JPG)

S6 Fig Expression of Connexin 40 and 43 at 1, 3 and 6 months. Immunohistochemical staining showing Connexin 40 (A and C) and 43 (B and D) expression in wt (left panels) and CHAPb Tg (right panels) left atria (A, B and upper panels of C and D) and right atria (lower panels of C and D) at one month (A and B) and 3 months of age (C and D) of age. qPCR analysis of Connexin 43 (E and F) expression in the left (E) and right (F) atrium at 6 months of age (wt, n = 3; Tg, n = 3; T-test: **,p<0.01). (G) Heart rate as determined by ECG in wild type and Tg mice (wt, n = 4; Tg n = 8; *t-test*: NS). Scale bars 50 μm.
(JPG)

S7 Fig Functional analysis of CHAPb Tg hearts. MRI measurements of the right ventricle of wt (n = 4, white bars) and CHAPb Tg (n = 5 black bars) animals at 6 months of age (A-D, *t*-test: *, p<0.05; **, p<0.01). ED volume (A), ES volume (B), ejection fraction (C) and cardiac output (D). ED (end diastolic), ES (end systolic) and LV (left ventricular).
(JPG)

S8 Fig Phosphorylation of sarcomeric proteins is not affected in 3-month-old CHAPb Tg mice left ventricles. (A) Representative image of a Pro-Q Diamond stained polyacrylamide gel that shows phosphorylated proteins (left panel). Representative image of a SYPRO Ruby total protein gel staining (right panel) (B) Quantification of phosphorylated MyBP-C, cTnT, cTnI and MLC2 in wildtype hearts (n = 5 mice) and CHAPb Tg hearts (n = 3 mice). No statistically significant difference in phosphorylation was found between wildtype and CHAPb Tg mice. (2-tailed unpaired *t-test*).
(JPG)

S9 Fig Expression of M-band protein myomesin is not affected in CHAPb Tg hearts. Hearts stained for CHAP (green) / myomesin (red), merge images are shown. CHAP is localized in the Z-disc of cardiomyocytes of wt (upper panels) hearts. In cardiomyocytes of CHAPb Tg (lower panels) CHAP also stained stress fibers (arrows), which did not stain for myomesin. Scale bars: 20 μm.
(JPG)

S10 Fig Ectopic pERM expression in CHAPb Tg hearts. Wt (upper panels) and CHAPb Tg (lower panels) hearts at 6 months of age stained for pERM (green) and alpha-actinin (red). Nuclei are stained blue, merge images are shown. Scale bars: 20 μm.
(JPG)

S1 Table PCR primers. (DOC)
