# Supplementary figures and images for: Z-disc protein CHAPb induces cardiomyopathy and contractile dysfunction in the postnatal heart
Source: PLoS One. 2017 Dec 5;12(12):e0189139. doi: 10.1371/journal.pone.0189139 (PMC5716575; doi:10.1371/journal.pone.0189139)

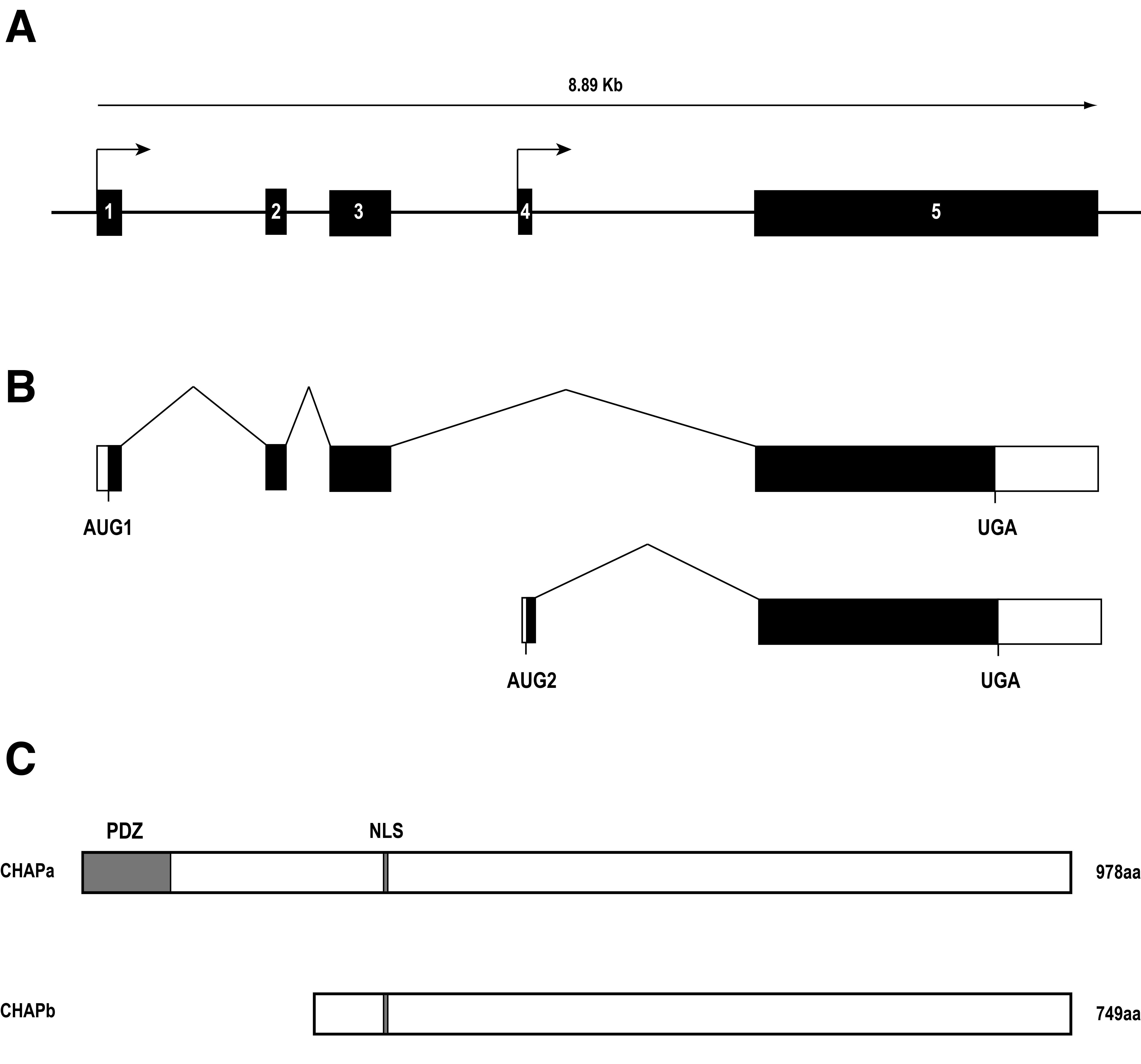

Supplement: S1 Fig — (A) Genomic organization of the mouse Chap gene on chromosome 14 (B) mRNA isoforms of CHAP resulting from differential usage of transcriptional start sites (C) CHAPa and CHAPb protein isoforms. (JPG) [file pone.0189139.s001.jpg]

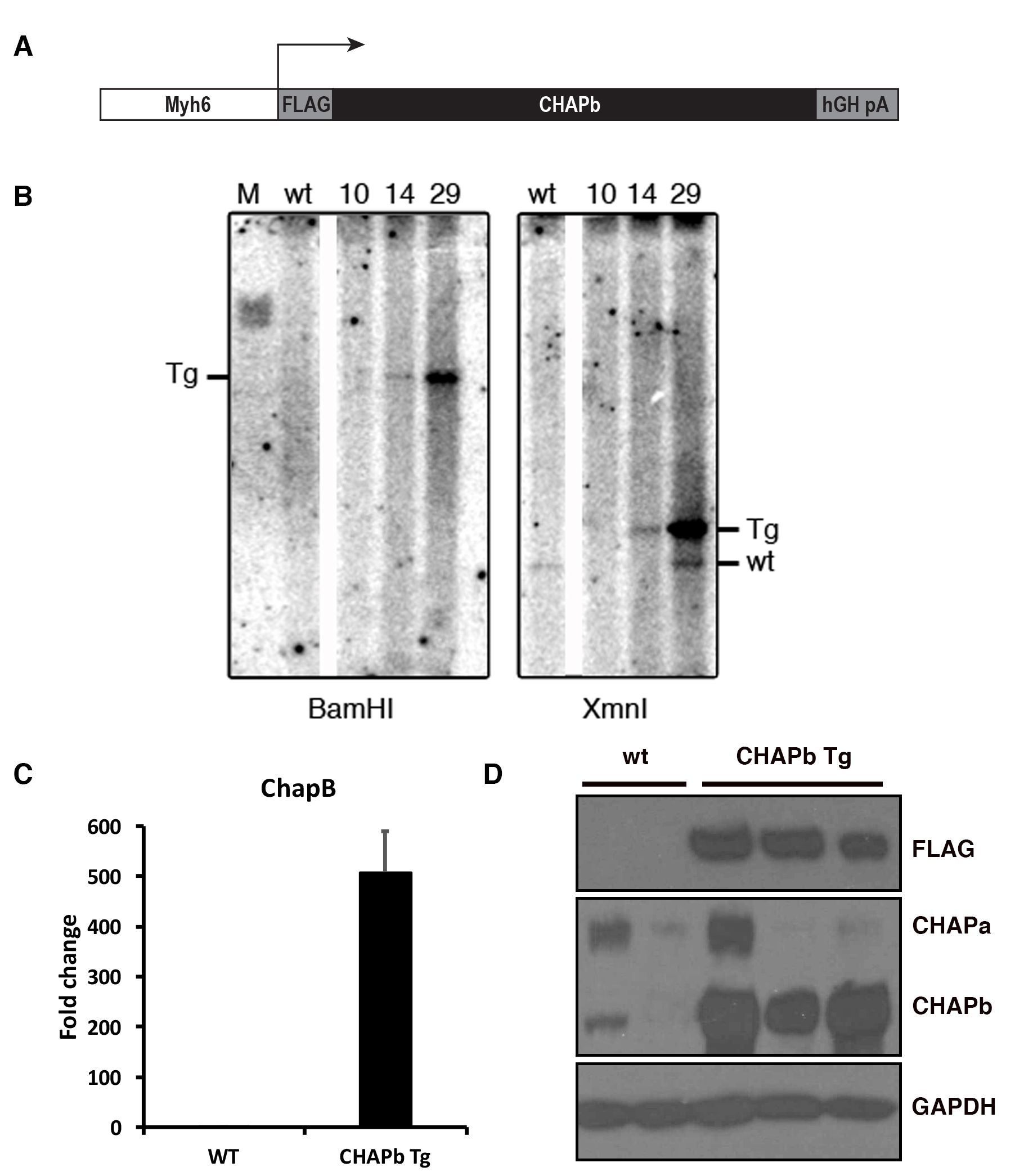

Supplement: S2 Fig — (A) Schematic overview showing the construct used to generate transgenic mice. FLAG-CHAPb cDNA is downstream of the α-MHC promoter and upstream a human Growth Hormone polyA signal. (B) Southern blot analysis of wild type and CHAPb Tg genomic DNA showing intermediate copy number in line 14 and high copy number in line 29, compared to wild type copy numbers. Hearts of CHAPb founder line 29 (left panels) and line 14 (right panels). (C) qPCR analysis of ChapB in wt (white bars, n = 6) and CHAPb Tg hearts (black bars, n = 3). Gapdh was used as internal control (D) Western blot showing CHAP and FLAG expression in wt (n = 2) and CHAPb Tg (n = 3) hearts. GAPDH was used as loading control. (JPG) [file pone.0189139.s002.jpg]

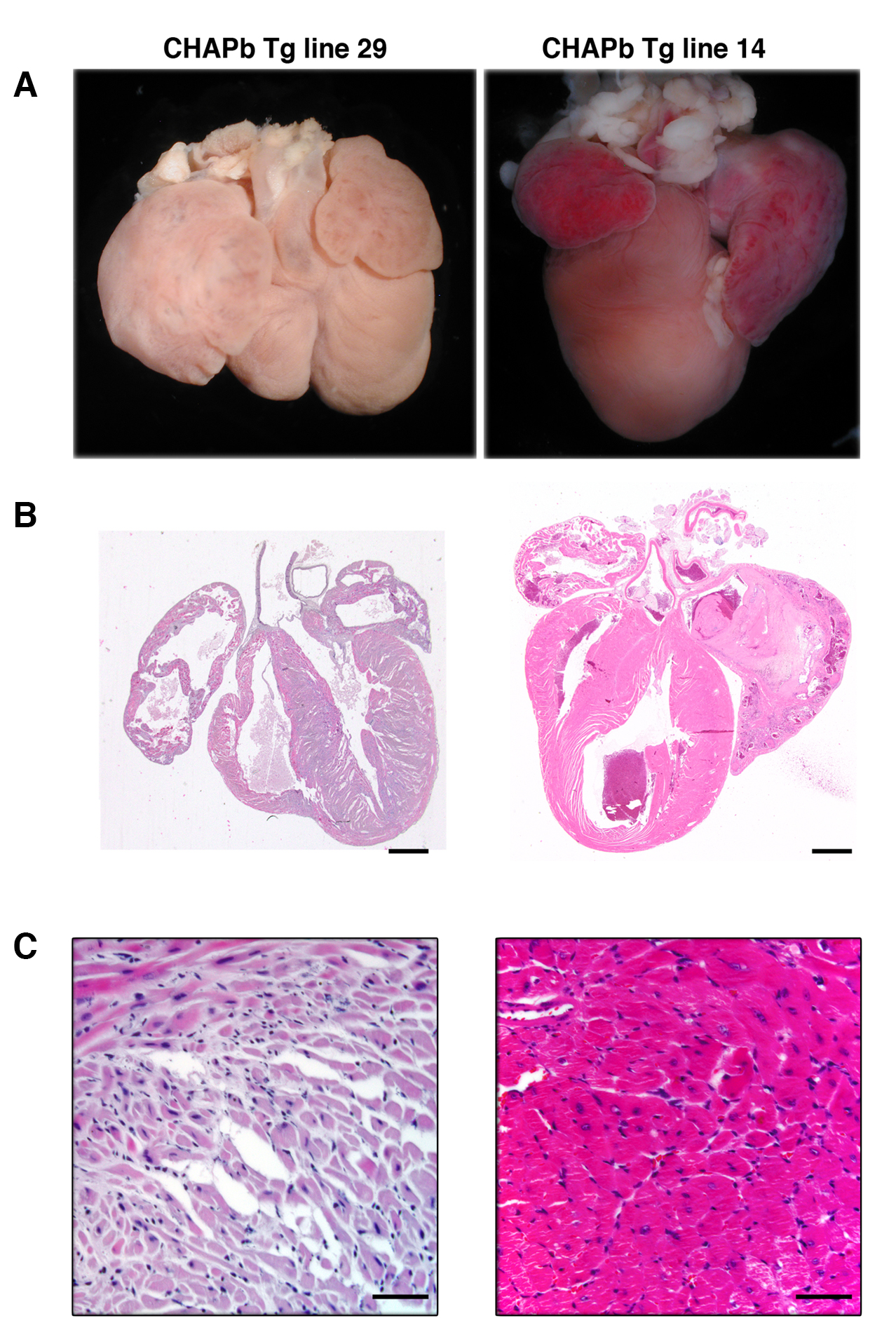

Supplement: S3 Fig — (A) Hearts showing enlarged atria and malformed ventricles. (B) HE stained overview section showing enlarged atria and thickened ventricles. (C) Higher magnification of the left ventricle. Scale bars in B 1 mm, in C 50 μm. (JPG) [file pone.0189139.s003.jpg]

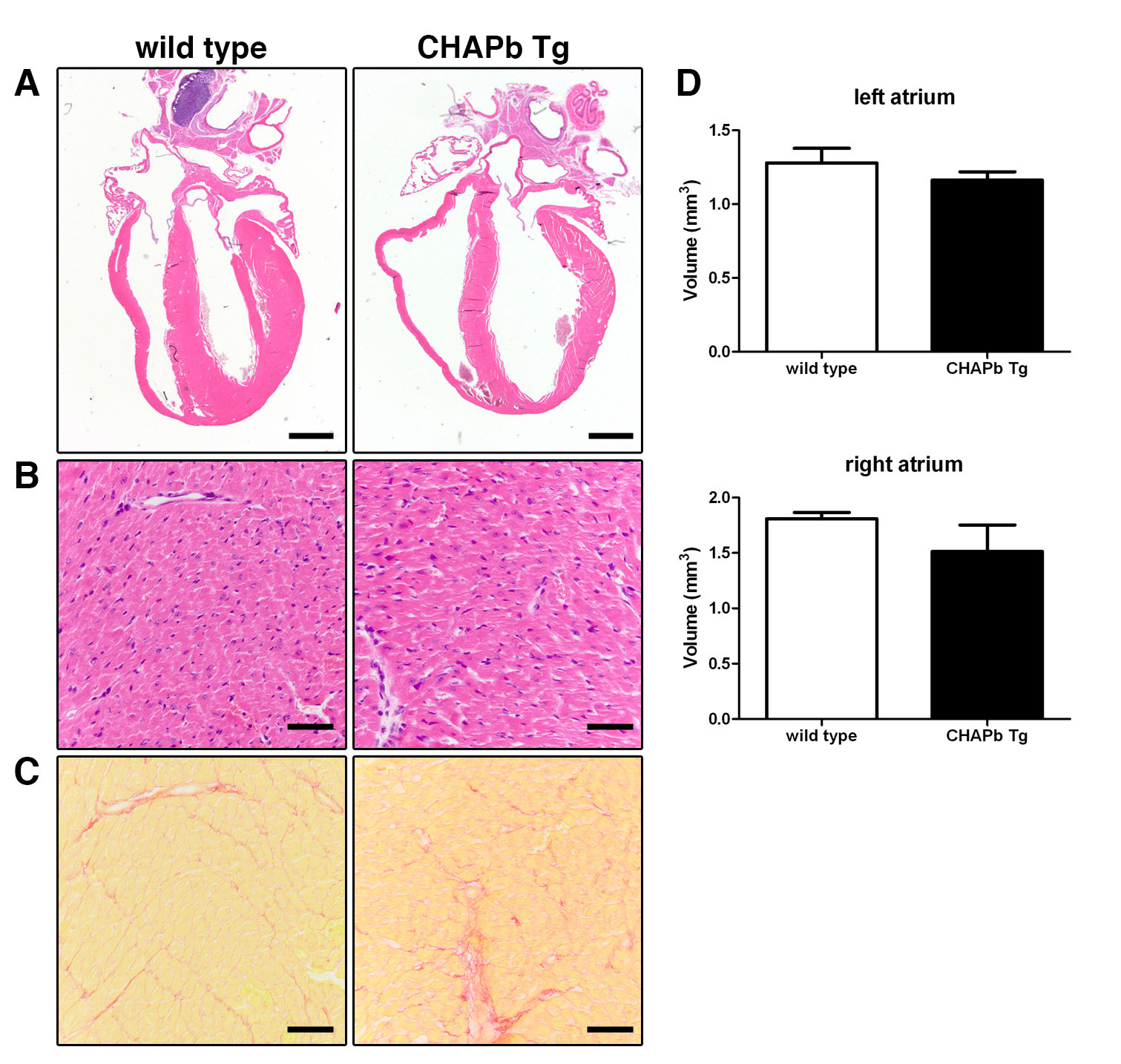

Supplement: S4 Fig — Wt (left panels) and CHAPb Tg (right panels) at 1 month of age (A-C). A) HE stained overview section. (B) Higher magnification of left ventricle. (C) Sirius red staining of the left ventricle. (D-E) Volume of the left atrium (D) and right atrium (E) in wt (n = 4, white bars) and CHAPb Tg (n = 4, black bars) hearts (t-test, NS). Scale bars 1 mm in A, 50 μm in B, C. (JPG) [file pone.0189139.s004.jpg]

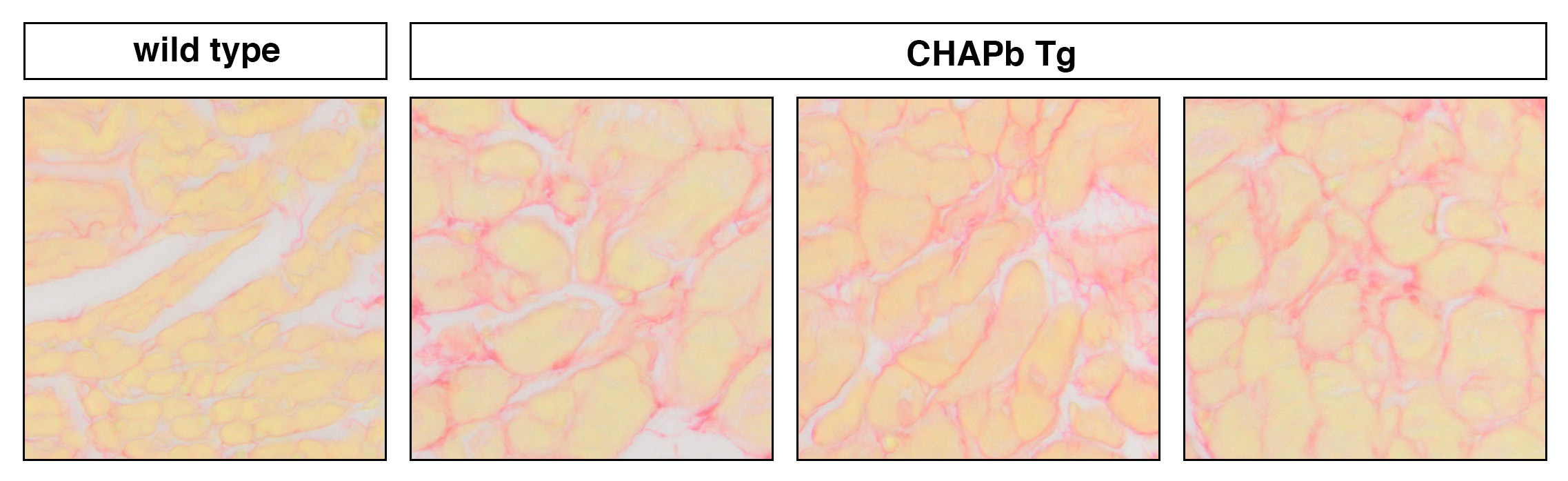

Supplement: S5 Fig — Sirius red staining on atrial sections of wild type (left panel) and CHAPb Tg mice shows increased interstitial fibrosis and increased myocytes size in CHAPb Tg atria. (JPG) [file pone.0189139.s005.jpg]

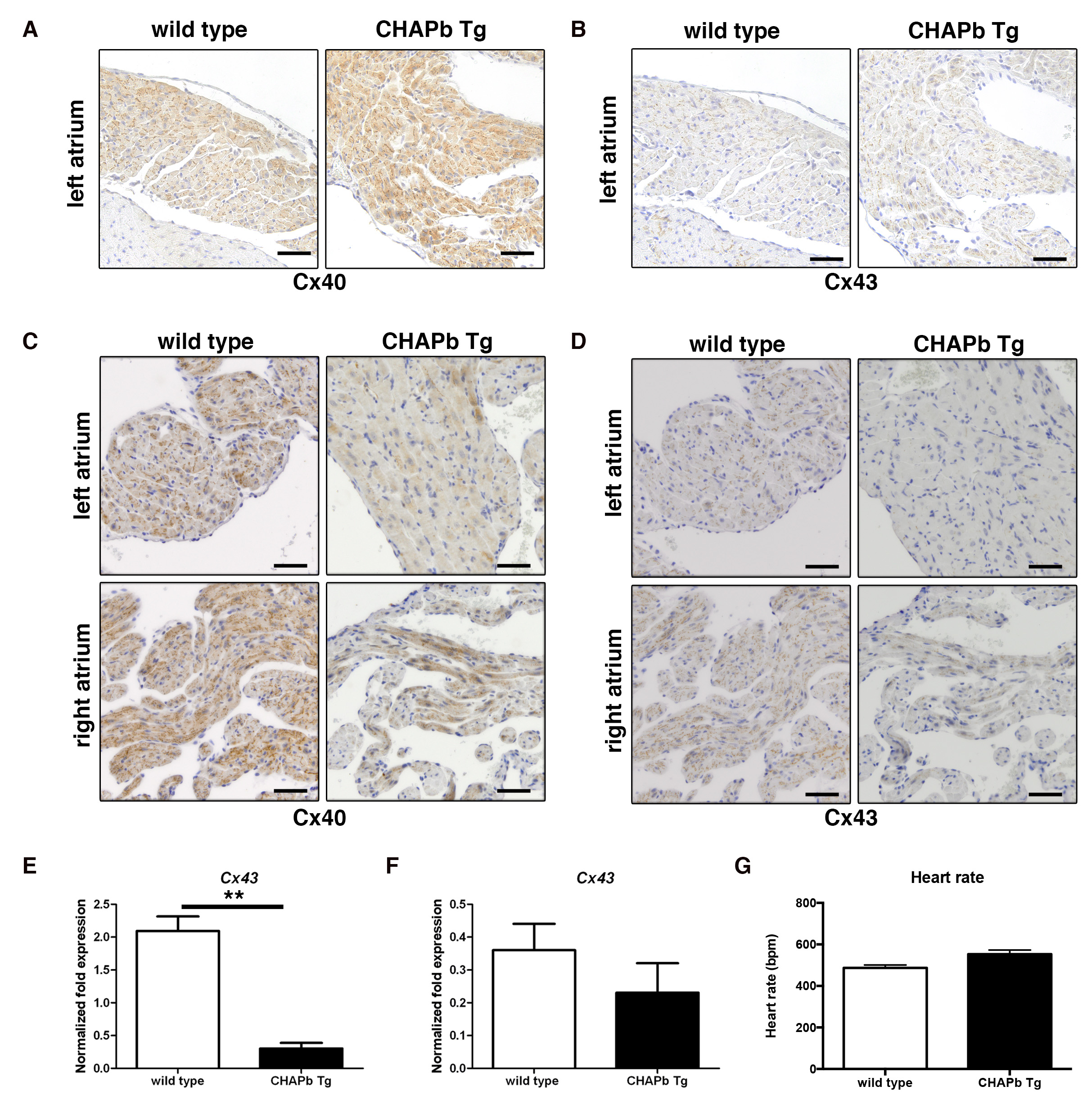

Supplement: S6 Fig — Immunohistochemical staining showing Connexin 40 (A and C) and 43 (B and D) expression in wt (left panels) and CHAPb Tg (right panels) left atria (A, B and upper panels of C and D) and right atria (lower panels of C and D) at one month (A and B) and 3 months of age (C and D) of age. qPCR analysis of Connexin 43 (E and F) expression in the left (E) and right (F) atrium at 6 months of age (wt, n = 3; Tg, n = 3; T-test: **,p<0.01). (G) Heart rate as determined by ECG in wild type and Tg mice (wt, n = 4; Tg n = 8; t-test: NS). Scale bars 50 μm. (JPG) [file pone.0189139.s006.jpg]

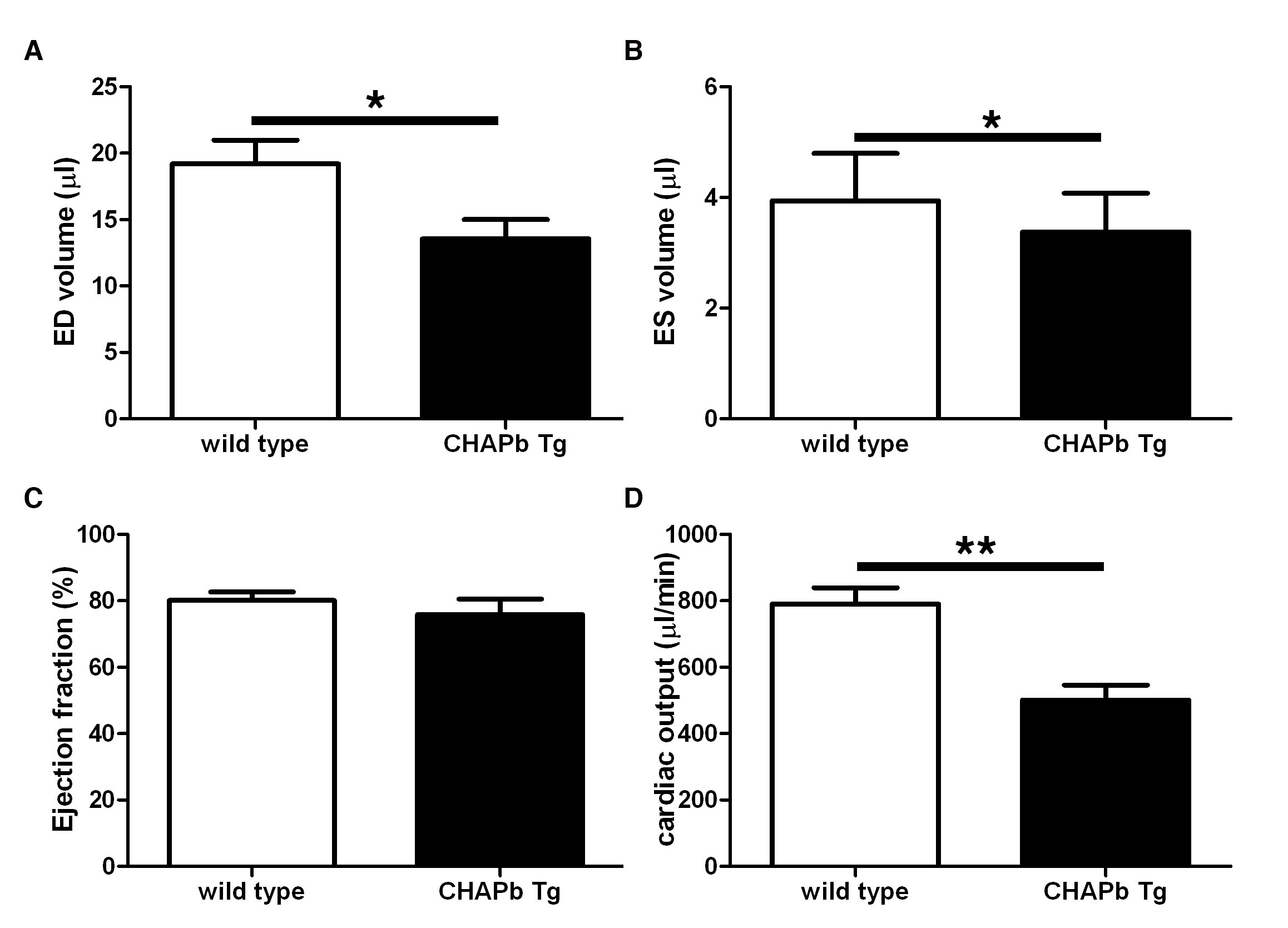

Supplement: S7 Fig — MRI measurements of the right ventricle of wt (n = 4, white bars) and CHAPb Tg (n = 5 black bars) animals at 6 months of age (A-D, t-test: *, p<0.05; **, p<0.01). ED volume (A), ES volume (B), ejection fraction (C) and cardiac output (D). ED (end diastolic), ES (end systolic) and LV (left ventricular). (JPG) [file pone.0189139.s007.jpg]

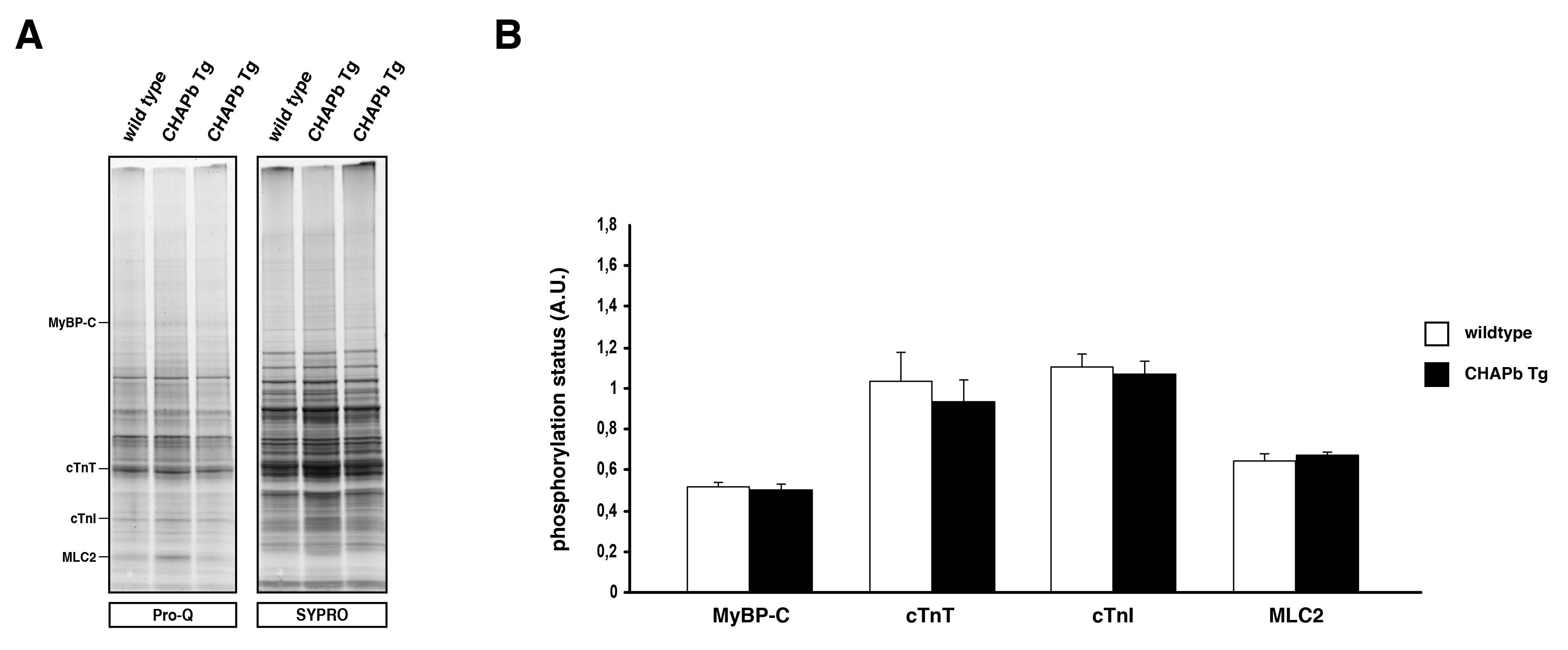

Supplement: S8 Fig — (A) Representative image of a Pro-Q Diamond stained polyacrylamide gel that shows phosphorylated proteins (left panel). Representative image of a SYPRO Ruby total protein gel staining (right panel) (B) Quantification of phosphorylated MyBP-C, cTnT, cTnI and MLC2 in wildtype hearts (n = 5 mice) and CHAPb Tg hearts (n = 3 mice). No statistically significant difference in phosphorylation was found between wildtype and CHAPb Tg mice. (2-tailed unpaired t-test). (JPG) [file pone.0189139.s008.jpg]
