# Supplementary material for: Z-disc protein CHAPb induces cardiomyopathy and contractile dysfunction in the postnatal heart
Source: PLoS One. 2017 Dec 5;12(12):e0189139. doi: 10.1371/journal.pone.0189139 (PMC5716575; doi:10.1371/journal.pone.0189139)
Supplement: S1 Table — (DOC) [file pone.0189139.s011.doc]

| **Gene name** | **Sequence** | **Melting temp. (^o^C)** | **Remarks** |
| --- | --- | --- | --- |
| *ChapA* | 5’-gaggaggtgcaggtcacatt-3’ | 58 |  |
|  | 5’-ctgaagagcctgggaaacag-3’ |  |  |
| *ChapB* | 5’-ccgccgcttcttaaacataa-3 | 58 | endogenous |
|  | 5’-ggctttaaagggccttgg-3’ |  |  |
| *ChapB* | 5’- CCAAGCCAGCTGTGACAAA -3’ | 58 | Endogenous + transgenic |
|  | 5’- CCGCCGCTTCTTAAACATAA -3’ |  |  |
| *Connexin40* | 5’- CTGGCTCACTGTCCTGTTCA -3’ | 60 |  |
|  | 5’- GCAACCAGGCTGAATGGTAT -3’ |  |  |
| *Connexin43* | 5’- TGGACAAGGTCCAAGCCTAC -3’ | 60 |  |
|  | 5’- ACAGCGAAAGGCAGACTGTT -3’ |  |  |
| *Connexin45* | 5’- AAGAGCAGAGCCAACCAAAA -3’ | 60 |  |
|  | 5’- CCCACCTCAAACACAGTCCT -3’ |  |  |
| *CollagenI* | 5’- GAGCGGAGAGTACTGGATCG -3’ | 60 |  |
|  | 5’- GTTCGGGCTGATGTACCAGT -3’ |  |  |
| *CollagenIII* | 5’- ACCAAAAGGTGATGCTGGAC-3’ | 60 |  |
|  | 5’- GACCTCGTGCTCCAGTTAGC -3’ |  |  |
| *Nppa* | 5’- GGGGGTAGGATTGACAGGAT -3’ | 60 |  |
|  | 5’- CAGAATCGACTGCCTTTTCC -3’ |  |  |
| *Nppb* | 5’- ACAAGATAGACCGGATCGGA -3’ | 60 |  |
|  | 5’- ACCCAGGCAGAGTCAGAAAC -3’ |  |  |
| *Myh7* | 5’- GAGCCTTGGATTCTCAAACG -3’ | 60 |  |
|  | 5’- CTTGCTACCCTCAGGTGGCT -3’ |  |  |
| *Serca2* | 5’- TACTGACCCTGTCCCTGACC -3’ | 60 |  |
|  | 5’- CACCACCACTCCCATAGCTT -3’ |  |  |
| *GAPDH* | 5’-GTTTGTGATGGGTGTGAACCAC-3’ | 58 | Reference gene |
|  | 5’- CTGGTCCTCAGTGTAGCCCAA -3’ |  |  |
| *H2A* | 5’-GTCGTGGCAAGCAAGGAG-3’ | 60 | Reference gene |
|  | 5’- GATCTCGGCCGTTAGGTACTC -3’ |  |  |
| *PGK* | 5’-tgagaaaggaagtgagctgtaaa-3’ | 52 | Reference gene |
|  | 5’-agattgccatgctgagtc -3’ |  |  |
